# Identification of Diagnostic Signatures and Immune Cell Infiltration Characteristics in Rheumatoid Arthritis by Integrating Bioinformatic Analysis and Machine-Learning Strategies

**DOI:** 10.3389/fimmu.2021.724934

**Published:** 2021-10-06

**Authors:** Rongguo Yu, Jiayu Zhang, Youguang Zhuo, Xu Hong, Jie Ye, Susu Tang, Yiyuan Zhang

**Affiliations:** ^1^ Department of Orthopedics, Fuzhou Second Hospital Affiliated to Xiamen University, Fuzhou, China; ^2^ School of Clinical Medicine, Yunnan University of Traditional Chinese Medicine, Kunming, China; ^3^ Department of Orthopedics, Fuzhou Second Hospital Affiliated to Xiamen University, Xiamen University, Xiamen, China

**Keywords:** rheumatoid arthritis, immune cells, diagnostic marker, bioinformatic analysis, machine-learning strategies

## Abstract

**Background:**

Rheumatoid arthritis (RA) refers to an autoimmune rheumatic disease that imposes a huge burden on patients and society. Early RA diagnosis is critical to preventing disease progression and selecting optimal therapeutic strategies more effectively. In the present study, the aim was at examining RA’s diagnostic signatures and the effect of immune cell infiltration in this pathology.

**Methods:**

Gene Expression Omnibus (GEO) database provided three datasets of gene expressions. Firstly, this study adopted R software for identifying differentially expressed genes (DEGs) and conducting functional correlation analyses. Subsequently, we integrated bioinformatic analysis and machine-learning strategies for screening and determining RA’s diagnostic signatures and further verify by qRT-PCR. The diagnostic values were assessed through receiver operating characteristic (ROC) curves. Moreover, this study employed cell-type identification by estimating relative subsets of RNA transcript (CIBERSORT) website for assessing the inflammatory state of RA, and an investigation was conducted on the relationship of diagnostic signatures and infiltrating immune cells.

**Results:**

On the whole, 54 robust DEGs received the recognition. Lymphocyte-specific protein 1 (LSP1), Granulysin (GNLY), and Mesenchymal homobox 2 (MEOX2) (AUC = 0.955) were regarded as RA’s diagnostic markers and showed their statistically significant difference by qRT-PCR. As indicated from the immune cell infiltration analysis, resting NK cells, neutrophils, activated NK cells, T cells CD8, memory B cells, and M0 macrophages may be involved in the development of RA. Additionally, all diagnostic signatures might be different degrees of correlation with immune cells.

**Conclusions:**

In conclusion, LSP1, GNLY, and MEOX2 are likely to be available in terms of diagnosing and treating RA, and the infiltration of immune cells mentioned above may critically impact RA development and occurrence.

## Introduction

Rheumatoid arthritis (RA) is recognized as a general chronic autoimmune connective tissue disease, which primarily covers the joints and eventually leads to chronic disability, joint destruction, and shortened life span (1–3). Rheumatoid arthritis affects 5 to 10 per 1,000 people (3). Ultimately, RA irreversibly damages joints, imposing a great adverse effect onto individual and society. Nevertheless, detection of RA at an early stage offers the opportunity for an effective treatment response, and this preclinical stage may be as short as a few months (4). At present, diagnosed rheumatoid factor and anticyclic citrullinated peptide antibody are serum biomarkers for the diagnosis of rheumatoid (5, 6). Yet early RA, particularly negative serum rheumatoid factor and anticyclic citrullinated antibodies, cannot easily be diagnosed for insufficient feasible biomarker. Therefore, finding novel and feasible biomarker is very important to early diagnose and treat RA.

Recently, increasing articles revealed that infiltration of immune cells critically impacts RA occurrence and progresses. For instance, RA exhibits a unique pattern of macrophage infiltration. The degree of macrophage infiltration in joint tissues and the level of monocyte-derived cytokines in serum show positive correlations to disease severity (7). CD8+ T cell exhibits anti-inflammation characteristic and is likely to contribute to the reduction of persistent autoimmune responses in rheumatoid joints (8, 9). B cells impact bone remodeling in RA (10). Nevertheless, the molecular system allowing different immune cells to impact RA occurrence and progresses should be clarified (11). For the mentioned reason, according to the aspect of immune systems, evaluating immune cells’ infiltration and ascertaining the distinctions within the infiltrating immune cells’ components are critical in elucidating RA molecular system and finding novel immunotherapeutic target. Cell-type identification by estimating relative subsets of RNA transcript (CIBERSORT) is a computational method for quantifying cell composition from tissue gene expression profiles obtained by RNA sequencing (12). Thus far, no studies have used CIBERSORT to analyze immune cell infiltration in whole blood of rheumatoid arthritis.

This study obtained RA microarray datasets in the GEO database to conduct investigations for differential expression gene. Besides, to screen and identify diagnostic markers of RA in depth, bioinformatics analysis, and machine-learning strategies were combined. Next, CIBERSORT was adopted for investigating the differences in whole-blood immune infiltrates in 22 immune cell subsets between RA and normal samples. Furthermore, the associations of diagnostic markers and infiltrating immune cells were investigated for gaining more insights into the molecular immune mechanisms involved in RA development.

## Materials and Methods

### Data Collection and Data Processing

Here, datasets received the search from the Gene Expression Omnibus (GEO) database (https://www.ncbi.nlm.nih.gov/geo/) with the keywords: “Arthritis, Rheumatoid” [MeSH Terms] OR “Arthritis, Rheumatoid” [All Fields] AND “Homo sapiens” [porgn: txid9606] AND “Expression profiling by array“ [All Fields]. The screening standards included the following: the microarray datasets referred to profiles of gene expressions with genome-wide of whole blood; the microarray datasets contained samples from RA and samples from healthy state; all included samples were not treated with drugs. Eventually, three datasets received the screening to achieve the in-depth investigation: GSE100191 (13), GSE17755 (14), and GSE93272 (15). The table of the clinical information for the samples of RA patients and healthy subjects is provided in **Supplementary Table 1**. According to the inclusion criteria, only RA and healthy samples were selected for further analysis, including 50 normal controls and 119 RA patients. Next, the present study conducted the data preprocessing based on RMA (16) (e.g., expression calculation, normalization, and background correction).

### Differential Expression Analysis

The present study adopted LIMMA (http://www.bioconductor.org/packages/release/bioc/html/limma.html) package for identifying DEGs through the comparison of the expression datasets of GSE100191 and GSE17755, and the volcano plot was drawn to present the differential expression of DEGs. DEGs with *P* < 0.05 and |log_2_ FC| > 1 were considered statistically significant. Next, the DEGs were further identified based on the “RobustRankAggreg” package in R to obtain robust DEGs. This method of Robust Rank Aggregation (RRA) can minimize the deviation and error between multiple datasets (17).

### Functional Correlation Analysis

For the exploration of the function and pathway of the identified feature gene, this study conducted the gene ontology (GO) and Kyoto Encyclopedia of Genes and Genomes (KEGG) pathway enrichment investigations with the use of the “clusterProfiler” package (18). *P <*0.05 was considered to show the statistical significance. In order to more intuitively clarify the gene expression level of significantly enriched functional pathways, gene set enrichment analysis (GSEA) was performed in R software (19).

### Screening and Verification of Diagnostic Markers

The four algorithms were adopted for screening of novel and key biomarkers for RA, including random forests (RF) (20, 21), least absolute shrinkage and selection operator (LASSO) logistic regression (22), support vector machine-recursive feature elimination (SVM-RFE) (23), and weighted gene co-expression network analysis (WGCNA) (24). This study adopted the random forest algorithm with R package “randomForest.” This study carried out LASSO logistic regression investigation with R package “glmnet,” and minimal lambda was considered optimal. This study conducted the featured gene selection with the RFE function within the caret package based on five-fold cross-validation. The SVM classifier was constructed using R package e1071 with five-fold cross-validation. WGCNA was performed by R package “WGCNA” (25). Then, this study selected the overlapping genes from the mentioned four classification models for further analysis. For the in-depth test of the efficacy of key biomarkers, the dataset of GSE93272 was combined with GSE100191 and GSE17755 as the validation set. It was assessed based on the investigation of receiver operating characteristic (ROC) curves (MedCalc software), and the area under the curve (AUC) was calculated for evaluating the predictive effect achieved by the algorithms. A two-sided *P* < 0.05 showed statistical significance.

### Quantitative PCR Analysis

A total of 34 whole blood samples (including 16 RA without drug treatments and 18 control samples with healthy state) were collected from Fuzhou Second Hospital affiliated to Xiamen University. The Ethical Committee of Fuzhou Second Hospital affiliated to Xiamen University approved this study, and the respective patient provided informed consent in a written form. All whole blood samples were immediately frozen in liquid nitrogen after the collecting process and stored at −80°C. The extraction of total RNA was performed with the use of Trizol reagent (TAKARA, Dalian, China). With a miRNA First Strand cDNA Synthesis Kit (Sangon, China), the reverse transcription of total RNA and miRNAs was performed. Besides, this study adopted the MicroRNAs qPCR Kit (Sangon, China) for examining miRNA and mRNA expressions, with the following primers: GAPDH (forward: 5′-GACAGTCAGCCGCATCTTCT-3′, reverse: 5′-ACCAAATCCGTTGACTCCGA-3′), LSP1 (forward: 5′-CTGTTAGCTTGGGAAGAGG-3′, reverse: 5′-ATAGCCCCTCTCAGATAGTC-3′), MEOX2 (forward: 5′-ATACTAGGGGAGATTCTCGC-3′, reverse: 5′-TAGGACTTTGGAGGGCTTAG-3′), and GNLY (forward: 5′-TCTGGTCCTAACTCTACTGG-3′, reverse: 5′-CAATCCTAGACAGTGTAGGC-3′) synthesized by Sangon Biotech. GAPDH was then handled as an internal reference. The relative expression was calculated using the 2−ΔΔCT method. *P* values < 0.05 showed statistical significance.

### Evaluation and Correlation Analysis of Infiltration-Related Immune Cells

The CIBERSORT website was used to filter 22 kinds of the immune cell matrix. According to *P* < 0.05, the immune cell infiltration matrix was obtained. The “ggplot2” package was used for PCA cluster investigation of the immune cell infiltration matrix. The present study adopted “corrplot” package for drawing the correlation heatmap for visualizing the correlation of 22 kinds of infiltrating immune cells. The “ggstatsplot” and “ggplot2” packages were adopted for analyzing the Spearman relationship between characteristic diagnostic markers and immune infiltrating cells and visualizing the result.

## Results

### Screening of DEGs in Different Datasets

**Figure 1:**
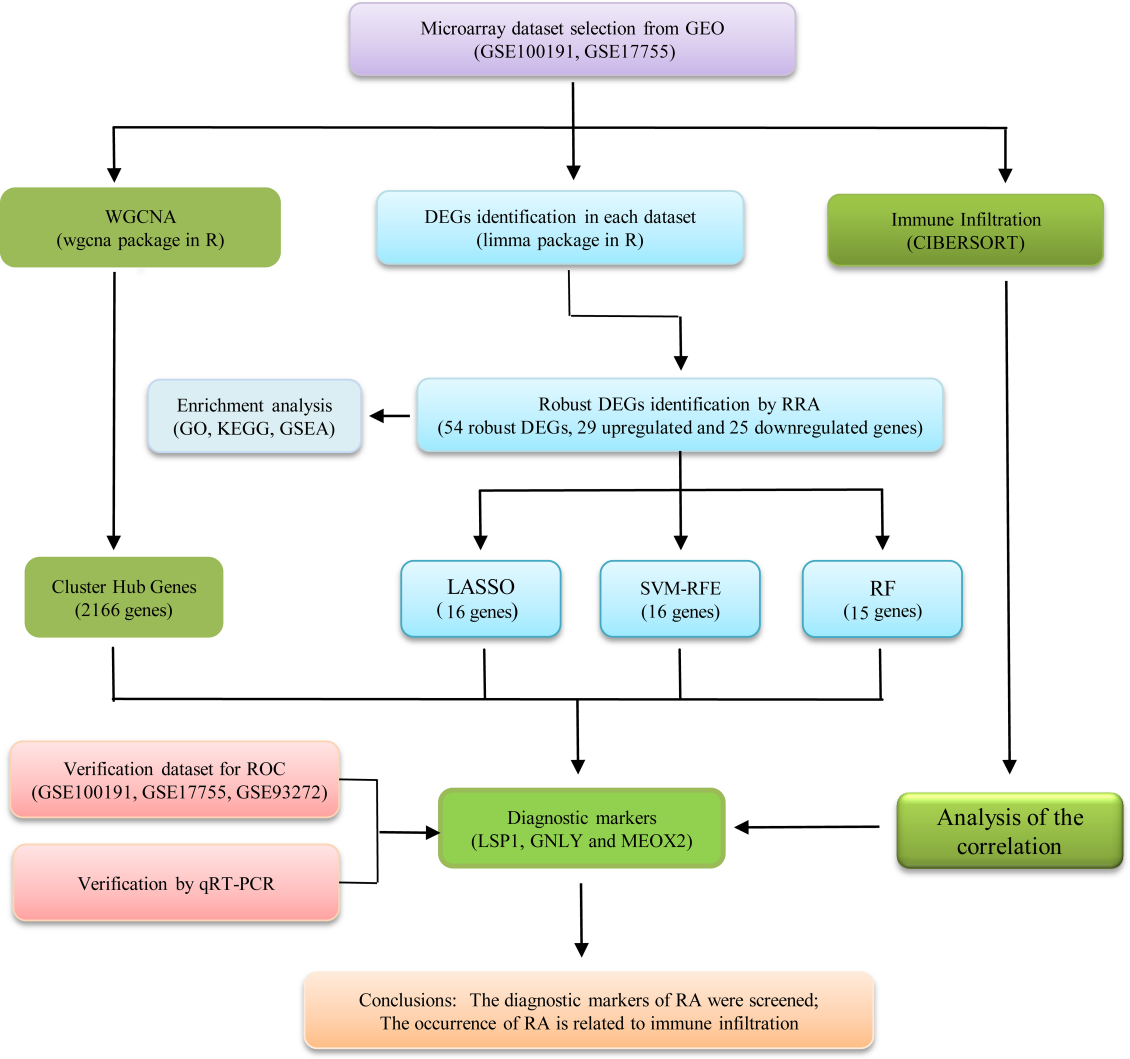
The flowchart of the analysis process.

**Figure 2:**
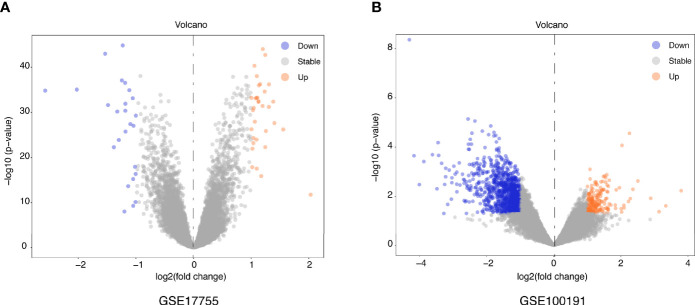
Volcano plots of DEGs distribution in GSE17755 **(A)** and GSE100191 **(B)**. Orange represented a high expression of robust DEG, while blue represented a low expression of robust DEG.

**Figure 1** illustrates a workflow of this study. There were 1,226 DEGs in GSE100191, including 207 upregulated and 1,019 downregulated genes (**Supplementary Table 2** and **Figure 2A**). Meanwhile, 58 DEGs were screened from the GSE17755 datasets, including 33 upregulated and 25 downregulated genes (**Supplementary Table 3** and **Figure 2B**). Next, 54 robust DEGs were screened in total with the RRA method (including 29 upregulated and 25 downregulated genes) (**Supplementary Table 4**).

### Functional Enrichment Analyses

**Figure 3:**
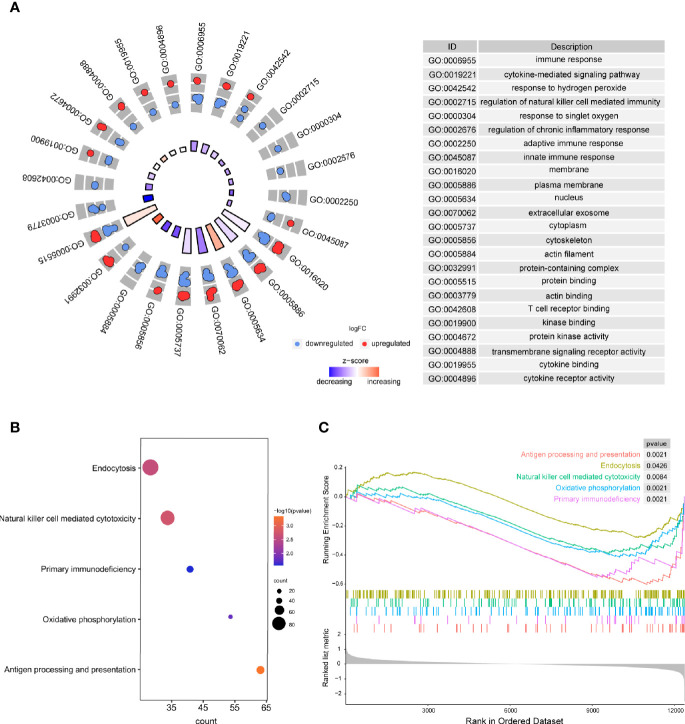
The results of functional enrichment analyses. **(A)** GO analyses results of DEGs; **(B)** Pathway analysis results of DEGs; **(C)** GSEA profiles depicting the five significant GSEA sets.

Based on the results of the present study, the significantly enriched biological processes included immune response, regulation of natural killer cell–mediated immunity, regulation of chronic inflammatory response, adaptive immune response, innate immune response, etc. (**Figure 3A**). Moreover, antigen processing and presentation, endocytosis, natural killer cell–mediated cytotoxicity, primary immunodeficiency, and oxidative phosphorylation were considered to be the most remarkably enriched pathways (**Figure 3B**), and GSEA results presented the enriched mainly pathways (**Figure 3C**). The above results suggest that the immune system is critical to RA.

### Screening and Verification of Diagnostic Markers

**Figure 4:**
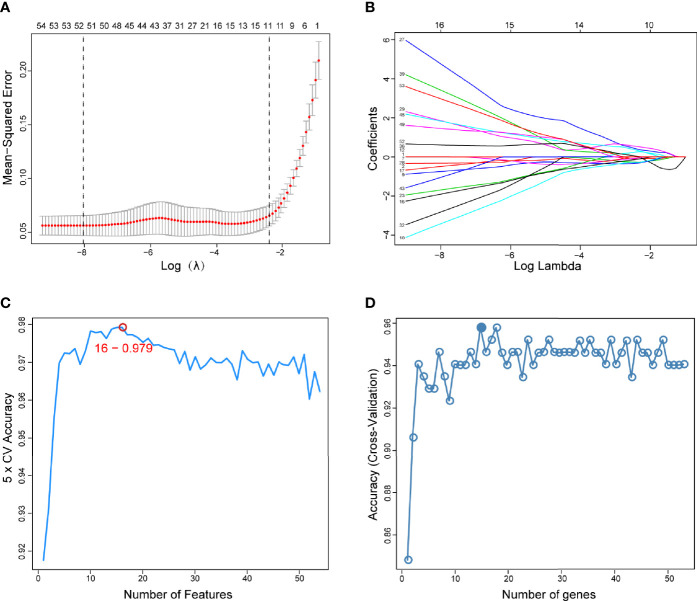
Screening of diagnostic markers *via* the comprehensive strategy. **(A)** Least absolute shrinkage and selection operator (LASSO) logistic regression algorithm to screen diagnostic markers; **(B)** Different colors represent different genes; **(C, D)** Based on support vector machine-recursive feature elimination (SVM-RFE) and random forest (RF) algorithm to screen biomarkers.

**Figure 5:**
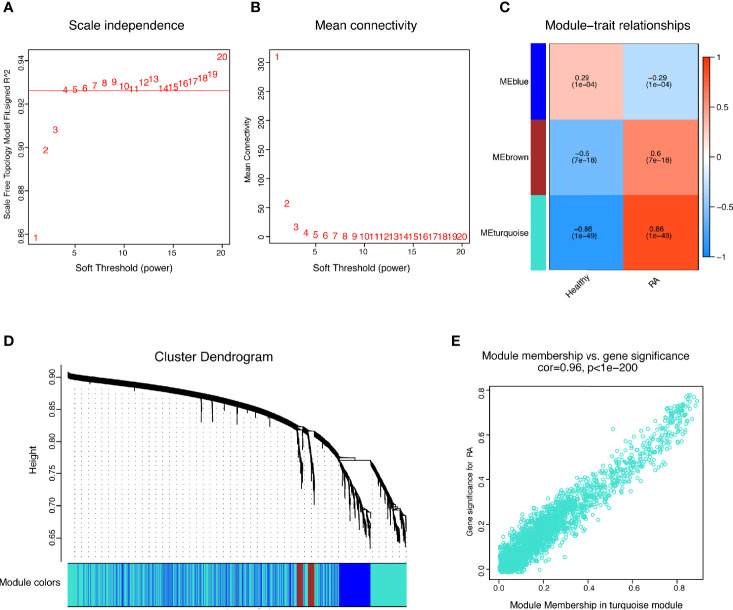
WGCNA revealed gene co-expression networks in the whole peripheral blood of 169 RA patients. **(A, B)** Analysis of the scale-free fit index and the mean connectivity for various soft-thresholding powers; **(C)** Relationships of consensus modules with samples. It contains a set of highly linked genes. Each specified color represents a specific gene module; **(D)** Clustering dendrogram of differentially expressed genes related to RA; **(E)** The gene significance for RA in the turquoise module (one dot represents one gene in the turquoise module).

**Figure 6:**
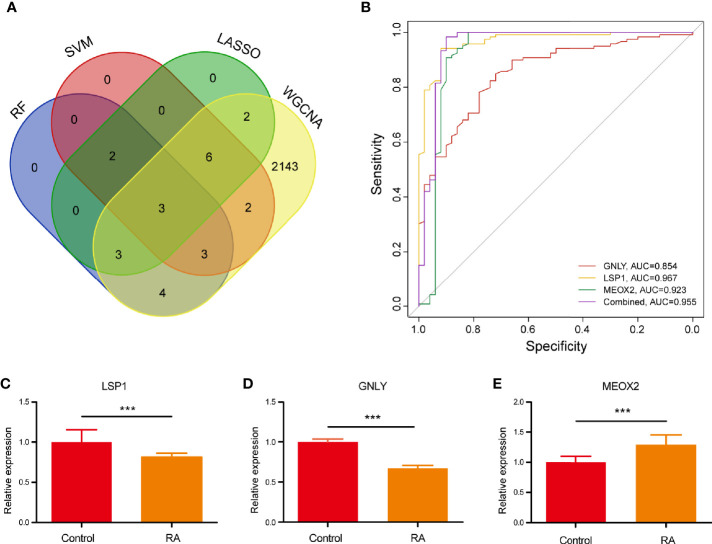
**(A)** Venn diagram showed the intersection of diagnostic markers obtained by the four algorithms. **(B)** The ROC curve of the diagnostic efficacy verification after fitting three diagnostic markers to one variable. **(C–E)** The miRNA expressions of potential diagnostic markers were validated by qRT-PCR. ***P < 0.001.

The present study adopted LASSO logistic regression algorithm to identify 16 key biomarkers from DEGs (**Figures 4A, B**). Sixteen genes were identified as key biomarkers from DEGs by the SVM-RFE algorithm (**Figure 4C**). Moreover, 15 genes were identified as vital biomarkers with RF algorithm (**Figure 4D**). When 0.92 acted as the correlation coefficient threshold, the soft-thresholding power was selected as 20 (**Figures 5A, B**). In accordance with WGCNA analysis, six remarkable co-expression modules were built. As indicated from the investigations of module-trait correlations, multiple modules were related to RA (**Figure 5C**), and the turquoise module was the most significant one, with 2,166 genes included in total (**Figures 5D, E**). LSP1, GNLY, and MEOX2 were overlapping genes by the four algorithms including one upregulated (MEOX2) and two downregulated (LSP1 and GNLY) genes (**Figure 6A**). The ROC curves of LSP1, GNLY, and MEOX2 revealed their probability as valuable biomarkers with AUCs of 0.967, 0.854, and 0. 923, respectively (**Figure 6B**), indicating that the three biological markers had a high accuracy of predictive value. The expression levels of the three biomarkers were examined by qRT-PCR in 34 whole blood samples. Three biomarkers (LSP1, GNLY, and MEOX2) were reported to be significantly dysregulated in RA compared with healthy samples. LSP1 and GNLY showed the significant downregulation, while MEOX2 showed a significant upregulation in RA (*P*<0.005) (**Figures 6C–E**), indicating that the results were reproducible and reliable.

### Infiltration of Immune Cells Results

**Figure 7:**
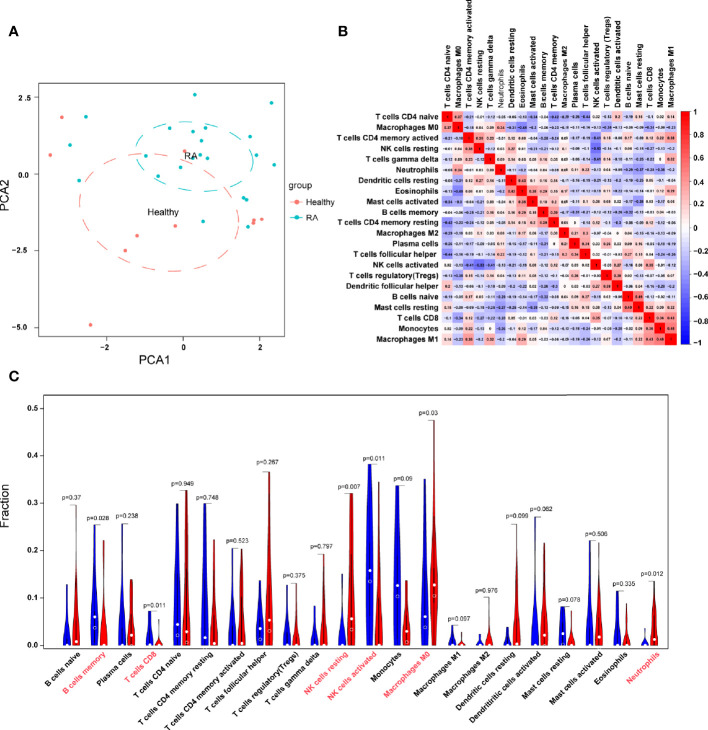
Evaluation and visualization of immune cell infiltration. **(A)** PCA cluster plot of immune cell infiltration between RA samples and control samples. **(B)** Heatmap of correlation in 22 types of immune cells. The size of the colored squares represents the strength of the correlation; red represents a positive correlation, and blue represents a negative correlation. Darker color implies stronger association. **(C)** Violin diagram of the proportion of 22 types of immune cells. The red marks represent the difference in infiltration between the two groups of samples.

With the CIBERSORT algorithm, we first summarized the results obtained from 50 normal controls and 119 RA patients. By PCA, the proportions of immune cells from the whole blood of RA patients and normal controls displayed distinct group-bias clustering and individual differences (**Figure 7A**). As indicated from the correlation heatmap of the 22 types of immune cells, eosinophils and M0 macrophages, resting T cells CD4 memory and naive T cells CD4, T cells follicular helper and naive T cells CD4, and activated NK cells and resting NK cells displayed a significant negative correlation, respectively. M1 macrophages and monocytes, M1 macrophages and T cells CD8, resting mast cells and naive B cells, eosinophils and resting dendritic cells displayed significant positive correlations, respectively (**Figure 7B**). In comparison with normal samples, RA samples generally contained a higher proportion of resting NK cells, neutrophils, whereas the proportions of B cells memory, T cells CD8, activated NK cells, and M0 macrophages were relatively lower (*P* < 0.05) (**Figure 7C**).

### Correlation Analysis Between Key Biomarkers and Infiltration-Related Immune Cells

**Figure 8:**
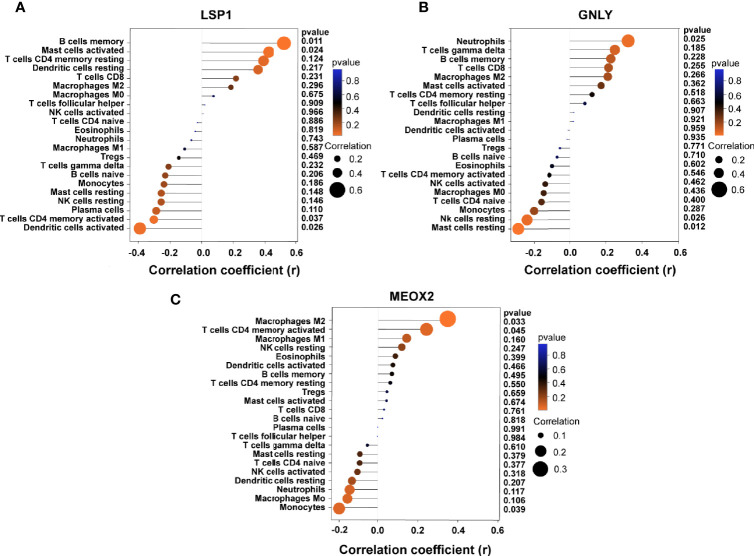
Correlation between diagnostic markers and infiltrating immune cells. **(A)** Correlation between LSP1 and infiltrating immune cells. **(B)** Correlation between GNLY and infiltrating immune cells. **(C)** Correlation between MEOX2 and infiltrating immune cells. The size of the dots represents the strength of the correlation between genes and immune cells; the larger the dots, the stronger the correlation, and the smaller the dots, the weaker the correlation. The color of the dots represents the *p*-value; the greener the color, the lower the *p*-value; and the red the color, the larger the *p*-value. *P* < 0.05 was considered statistically significant.

Based on the results of correlation analysis, LSP1 displayed a positive correlation with memory B cells (r = 0.512, p = 0.011) and activated mast cells (r = 0.423, p = 0.024) and showed a negative correlation with activated dendritic cells (r = −0.382, p = 0.026) and activated T cells CD4 memory (r = −0.341, p = 0.037) (**Figure 8A**). GNLY showed a positive correlation with neutrophils (r = 0.321, p = 0.025) and showed a negative correlation with resting mast cells (r = −0.292, p = 0.012) and resting NK cells (r = 0.242, p = 0.026) (**Figure 8B**). MEOX2 showed a positive correlation with M2 macrophages (r = 0.382, p = 0.033) and activated T cells CD4 memory (r = 0.282, p = 0.045) and showed a negative correlation with monocytes (r = 0.202, p = 0.039) (**Figure 8C**).

## Discussion

RA primarily features chronic synovitis, systemic inflaming process, as well as the arrival of autoantibodies, leading to chronic inflammation, joint damage, and dysfunction of other vital organs. Moreover, according to existing articles, infiltration of immune cells noticeably impacts on RA’s occurrence and progresses (8, 26). Therefore, it is of profound significance to search for particular diagnosis marker and analyze the infiltration patterns pertaining to RA immune cells in terms of facilitating RA cases’ prognosis. Here, an attempt was made for finding diagnosis marker pertaining to RA and delving into the effect exerted by infiltration of immune cells within RA.

In our study, we identified 54 robust DEGs, covering 29 risen and 25 declined DEGs, by comparing genes expressed in RA and normal samples. Afterwards, the DEGs underwent the annotation based on function-related enrichment study. The mentioned genes displayed tight associations to immune responses and inflaming signals (e.g., immune responses, regulation of natural killer cell–mediated immunity, responses to singlet oxygen, regulation of chronic inflammatory response and adaptive immune response). KEGG channels undergoing the enriching process covered endocytosis, cytotoxicity under the mediation of natural killer cell, antigen-presenting process and processing, primary immunodeficiency, and oxidative phosphorylation. Based on GO and KEGG enrichment study, RA achieved robust immune activating process and immune cell involvements, largely causing RA synovial inflaming process, thereby inducing arthralgia and arthritis. Generally, arthralgia and arthritis refer to the major RA clinically related reflections (3).

The model of random forest (RF) refers to a non-parametric approach to achieve the classifying process under the supervision (21). RF covers decision tree respectively originating from data subdivided set. The present work conducted the training and analysis for one RF classifying model for identifying descriptors that could discriminate RA from general sample. LASSO logistic regression, one machine-learning algorithm, determined variables by searching for λ under the smallest probability of classification error (22). SVM Recursive Feature Elimination (SVM-RFE) refers to an approach for machine learning and achieves extensive applications to rank features and to select the significant ones for classification (23). WGCNA refers to an approach for investigating gene expressing modes within sample. Gene exhibiting consistent expressing modes underwent the clustering process, and the relations of the module with particular trait or phenotype can be determined (24). We integrated the four different algorithms, each of which had its own inherent characteristics. Finally, LSP1, GNLY, and MEOX2 were selected and were accurate for in-depth verifications here, which suggested that our prediction exhibited the feasibility by the integration strategy.

Lymphocyte-specific protein 1 (LSP1) is capable of encoding intracellular F-actin-binding protein (27), achieving the expressions within endothelial cell, macrophage, neutrophil, and lymphocyte and regulating neutrophils’ movement, fibrinogen matrix protein adhesion, and transendothelial migration (28). The F-actin-bound cytoskeleton protein LSP1 has been identified as a regulator of neutrophil chemotaxis during inflammation (29, 30). Hwang et al. reported that cases with RA had reduced LSP1 expression in peripheral blood T cell, but improved migration ability, indicating that defects in the LSP1 signaling pathway lowered T-cell activation threshold (cell migrating process) in RA cases (27). LSP1 regulates a variety of biological processes in immune cells. However, immune cell, largely comprising macrophage, T cell, and B cell, to be autoimmune disease, critically impacts the pathogenesis of RA. Granulysin (GNLY), a member of the saponin family, has a location within the cytotoxic granules pertaining to T cells and is released in response to antigen stimulation. GNLY is a cytotoxic granuloprotein secreted by cytotoxic T lymphocytes and natural killer cells (31, 32). Although many studies have evaluated serum GNLY as a biomarker in cases with solid or hematological malignancies (33, 34), few studies have reported serum GNLY concentrations in RA cases (35). Mesenchymal homobox 2 (MEOX2) encodes a member of a non-aggregated, divergent, tentacle-like homobox gene subfamily. The MEOX family includes two homologous domain proteins, MEOX1 and MEOX2, which have 95% sequence homology in the homologous domain and are required for the normal development of bone and muscle in mouse embryos (36). MEOX2 expression is inhibited by zinc finger binding protein (37), and the abnormal expression of zinc finger protein displays a tight relation with RA occurrence and progresses (38, 39). Accordingly, this work infers that MEOX2 is likely to critically impact RA progresses. Considering the above findings, LSP1, GNLY, and MEOX2 are likely to impact RA progresses and act as diagnosis markers, whereas a large number of clinically related articles are further required for the verification of the diagnosis significance for LSP1, GNLY, as well as MEOX2.

To more specifically examine effects exerted by infiltration of immune cell in RA, the present study applied CIBERSORT for assessing the immune infiltrating process within RA. The infiltration of resting NK cells and neutrophils increased, while the infiltration of B cell memory, T cell CD8, activated NK cells, and M0 macrophages decreased, probably showing associations with RA occurrence and progresses. It is well known that B cells are a vital part pertaining to human adaptive immunity, whereas these cells under RA become a possible factor in RA pathogenesis (40). Local synthesizing process for cytokine (e.g., IL-1α, IL-23, IL-12, IL-6, and TNF-α) under the induction from local autoreactive B cell was suggested to impact pathology-associated RA cells, triggering bone injury, inflammation, and immune disorder (41, 42). It is currently evidenced that CD4+ T helper cells impact the pathogenesis of RA largely *via* the secreting process for cytokine and chemokine. Type 1 T-helper cells achieve the significant activation within RA and secrete pro-inflammation cytokines (e.g., IFN-γ, IL-2, and TNF-α) (43). CD8+ T cells exhibit anti-inflammation characteristic and are likely to contribute to the reduction of ongoing autoimmune responses in rheumatoid joints (8). CD56+ NK cells were overexpressed and produced higher levels of IFN-γ in inflammatory joints compared with NK cell of peripheral blood (44). Nevertheless, NK cell’s exact mechanism continues to be unclear. Under normal conditions (45), most macrophages exist in the tissue in a resting state. However, in inflammatory joints, they conduct the regulation of the secreting process for pro-inflammatory cytokine and injury-associated enzyme under the relation to the inflaming response and afterwards trigger joint destructing process (45). Although this has been mentioned many times, further research into the molecular mechanisms and functions of immune cell infiltration in rheumatoid arthritis is urgently needed.

Based on the investigation of the correlations of immune cell and diagnostic signatures, LSP1 suggested a positive correlation with memory B cell and mast cell under the activation and negative correlations to activated dendritic cell and activated T cell CD4 memory. GNLY showed positive correlations to neutrophils and showed negative correlations to resting mast cell and resting NK cell. MEOX2 showed positive correlations to M2 macrophage and activated T cell CD4 memory and a negative correlation with monocytes. Interestingly, a study reported found that cases with RA achieved declining LSP1 expressing state within peripheral T cell with improved migratory ability, thereby demonstrating that defects within LSP1 signaling lead to the decline of T-cell activation threshold (46). Kulkarni et al. reported that LSP1 underwent the interacting process with the interferon-inducible protein inside dendritic cell for facilitating surface-bound HIV-1 endocytosis and early endosome forming processes (47). Granulysin refers to one protein in the granules of natural killer cell and human cytotoxic T lymphocyte, exhibiting cytolysis activity against tumor and microbe (32), whereas there is no information concerning the mechanisms involved in RA. Due to a relatively small amount of research, the sophisticated interacting processes of gene and immune cell should be investigated in depth based on the mentioned assumption.

New science approaches (e.g., RF, LASSO logistic regression, WGCNA, and SVM-RFE algorithm) were used for identifying RA diagnosis-related markers. Besides, CIBERSORT was used for investigating infiltration of immune cells. Nevertheless, this study is subject to some limits. The CIBERSORT investigation complies with confined genetic information probably deviating from cellular heterogeneity interacting process, disease-induced diseases, or phenotypic plastic property. Moreover, this study indicates a 2^nd^ mining and investigation for existing datasets. Though the results of several existing studies show no consistency with the result of this analysis, whether the results here are reliable should receive in-depth verification by experiments with large samples.

## Conclusions

In brief, this study reported that LSP1, GNLY, and MEOX2 refer to diagnostic markers of RA. This study also reported that resting NK cells, neutrophils, memory B cells, T cells CD8, activated NK cells, and M0 macrophages are likely to participate in the occurrence and progress of RA. In addition, LSP1 was significantly associated with memory B cells, activated mast cells, activated dendritic cells, activated T cells CD4 memory; GNLY was significantly associated with neutrophils, resting mast cells, resting NK cells; MEOX2 was significantly associated with M2 macrophages, activated T cells CD4 memory, monocytes. The mentioned immune cells are likely to critically impact RA development, and the in-depth exploration of the immune cells is likely to ascertain the targets in immunotherapy and help optimize immunomodulatory therapy for RA patient.

## Data Availability Statement

The datasets presented in this study can be found in online repositories. The names of the repository/repositories and accession number(s) can be found in the article/**Supplementary Material**.

## Ethics Statement

The studies involving human participants were reviewed and approved by The Ethical Committee of Fuzhou Second Hospital affiliated to Xiamen University. The patients/participants provided their written informed consent to participate in this study.

## Author Contributions

YYZ and RY: conception and design of the study and funding acquisition. RY, JZ, and YGZ: data acquisition, bioinformatics analysis, and drafting and critical revision of the manuscript. RY, XH, ST, and JY: visualization and validation. All authors contributed to the article and approved the submitted version.

## Funding

This study was supported by the Project of innovation platform for Fuzhou Health and Family Planning Commission (Nos. 2017-S-wp1 and 2018-S-wp3).

## Conflict of Interest

The authors declare that the research was conducted in the absence of any commercial or financial relationships that could be construed as a potential conflict of interest.

## Publisher’s Note

All claims expressed in this article are solely those of the authors and do not necessarily represent those of their affiliated organizations, or those of the publisher, the editors and the reviewers. Any product that may be evaluated in this article, or claim that may be made by its manufacturer, is not guaranteed or endorsed by the publisher.

## Supplementary Material

Supplementary Table 1 The characteristic of three datasets

Supplementary Table 2 The list of differentially expressed genes of GSE100191.

Supplementary Table 3 The list of differentially expressed genes of GSE17755

Supplementary Table 4 Fifty-four robust DEGs were screened with the RRA method from the two lists of differentially expressed genes

## Abbreviations

RA, rheumatoid arthritis; ROC, receiver operating characteristic; CIBERSORT, cell-type identification by estimating relative subsets of RNA transcript; GEO, Gene Expression Omnibus; RRA, Robust Rank Aggregation; GO, gene ontology; KEGG, Kyoto Encyclopedia of Genes and Genomes; GSEA, gene set enrichment analysis; RF, fandom forests; LASSO, least absolute shrinkage and selection operator; SVM-RFE, vector machine-recursive feature elimination; WGCNA, weighted gene co-expression network analysis; AUC, area under the curve; LSP1, lymphocyte-specific protein 1; GNLY, granulysin; MEOX2, mesenchymal homobox 2.
