# Supplementary material for: Identification of Diagnostic Signatures and Immune Cell Infiltration Characteristics in Rheumatoid Arthritis by Integrating Bioinformatic Analysis and Machine-Learning Strategies
Source: Front Immunol. 2021 Oct 6;12:724934. doi: 10.3389/fimmu.2021.724934 (PMC8526926; doi:10.3389/fimmu.2021.724934)
Supplement: Supplementary Table 1 — The characteristic of three datasets [file Table_1.docx]

| **The characteristic of three datasets** | | | | | | | |
| --- | --- | --- | --- | --- | --- | --- | --- |
| Datasets | RA | | | Normal | | | Platform |
|  | Female | Male | Age(years) | Female | Male | Age(years) |  |
| GSE100191 | 5 | 2 | 44.0±8.8 | 5 | 0 | 34.4±8.4 | GPL13497 |
| GSE17755 | 93 | 19 | 51.9±11.1 | 24 | 29 | 31.7±12.9 | GPL1291 |
| GSE93272 | 205 | 27 | 57.2±14.7 | 38 | 5 | 42.4±9.3 | GPL570 |
| RA Rheumatoid arthritis | | | | | | | |
