# Supplementary material for: Identification of Diagnostic Signatures and Immune Cell Infiltration Characteristics in Rheumatoid Arthritis by Integrating Bioinformatic Analysis and Machine-Learning Strategies
Source: Front Immunol. 2021 Oct 6;12:724934. doi: 10.3389/fimmu.2021.724934 (PMC8526926; doi:10.3389/fimmu.2021.724934)
Supplement: Supplementary Table 2 — The list of differentially expressed genes of GSE100191. [file Table_2.pdf]

Gene	logFC	P.Value
CPB2	2.258597498	0.0000274
PANK4	2.035822922	0.0000851
TAS2R1	1.084093314	0.000792951
OTP	1.581643309	0.001320439
CITED1	1.518049275	0.001476121
ITPKB	1.480965932	0.001566372
RASGEF1B	1.547565423	0.001740295
PLSCR5	1.087256557	0.001962924
TXNRD1	1.657426556	0.002210472
AHRR	2.508125333	0.002356124
PCGEM1	1.466254166	0.002480953
CPNE6	1.410810392	0.00255386
NFML	1.261808754	0.002586914
CAF1	1.228684561	0.002655072
SLC22A17	1.552983972	0.002662941
CLC	1.266683488	0.002988659
TMPRSS7	1.242443277	0.003336436
MAT2B	1.29939276	0.003432668
LPHN2	1.593882606	0.003468906
PRKAA1	1.132884689	0.003579779
MYEF2	1.63073776	0.003914372
PVRL1	1.007137124	0.003955581
SYS1	1.395908592	0.004391934
PTGFRN	1.959181415	0.004462568
KLF9	1.125936016	0.004735541
CD99L2	1.164643179	0.00494351
FOS	2.361477974	0.005151697
FOXJ2	1.481659891	0.005329381
ZNF646	1.495533992	0.005374688
ADORA1	1.441470109	0.005451508
DUSP1	1.864217354	0.005455016
AFAP1	1.445372345	0.005805655
EDA2R	1.371740411	0.005861041
EGR1	3.797954328	0.005890302
RBM3	1.284803681	0.00589614
PIK3CB	1.11709621	0.006157529
LOC149837	1.590568983	0.006317999
ZFP36	1.392575868	0.006574782
FONG	1.612144354	0.006658127
C22orf24	1.11844302	0.006766518
GPR78	1.178208175	0.00687593
OC90	1.349246888	0.007730924
NPHS2	1.374543917	0.007778251
EI24	1.412950986	0.007820294
STXBP6	1.036779854	0.007821056
PRSS33	1.057592432	0.007945755
C22orf35	1.05105876	0.007981715
S100A12	1.073552081	0.00863871
MC5R	1.30781931	0.008696971
LHFPL5	1.263574117	0.00893799
SLC25A26	1.343061508	0.009099441
CTSB	1.180500282	0.009567485
GPX1	1.11680107	0.009882169
C1orf216	1.02702026	0.010028556
ETS1	1.395422427	0.010028914
TMEM176B	1.29815403	0.01063388
OR2A9P	1.277771815	0.011245096

UBE2D1	1.165682904	0.011547801
IL3R2	1.044431991	0.011581348
CBLL1	1.130633018	0.011798671
EGR2	2.889423033	0.011969837
PRDX3	1.578948078	0.012030539
CD44	1.071663947	0.012173674
ATP6V1E1	1.317938773	0.012304079
FOXM1	2.281168956	0.012313736
CD9	1.553648874	0.012502125
A2ML1	1.144565365	0.012530586
GLUL	1.081017422	0.012752449
CRISPLD2	1.146678438	0.012892692
SEC31A	1.015381059	0.01306704
LOC643529	1.152670202	0.013427106
MAPK3	1.036460777	0.013610329
C15orf17	1.278129606	0.014369114
MB21D2	1.369581676	0.014620346
C22orf25	1.122511152	0.014726737
C8orf31	1.153305779	0.01488454
GABRD	1.055652976	0.014901854
CX3CL1	1.141030056	0.015102367
KANK2	1.372725001	0.015204284
CD55	1.008065031	0.01524093
MEOX2	1.517558791	0.015352696
SLC3A2	1.186884917	0.015429103
KIAA1467	1.144640912	0.015868734
D21S2090E	2.229152032	0.016129427
DPYSL4	1.434408159	0.016143677
RIN2	1.096848197	0.016449517
RRAGD	1.182268338	0.016612481
IL12RB2	1.136087967	0.016648466
STC1	1.275534049	0.016685032
HMGN4	1.091123921	0.016875056
OLFM3	1.426943924	0.017043142
TAGAP	1.234284194	0.017495256
MAN1A1	1.135806991	0.017734034
LRTM1	1.145745832	0.017791779
MRPS36	1.14264422	0.018137272
HBEGF	1.127468194	0.018848243
F5	1.134299173	0.018862575
VCL	1.230338206	0.019675174
KRTAP15-1	1.272462914	0.020023681
RNF157	1.660036876	0.020087209
MXD4	1.362664296	0.020180945
SERD	1.516776823	0.020329128
EMP1	1.318744906	0.020672564
ZNF597	1.094360683	0.020835932
ITLN1	1.338191376	0.020972994
C19orf22	1.314267764	0.021538769
FAM117A	1.033712867	0.021539174
NOC4L	1.06239982	0.021952742
CDGH	1.24070818	0.022023962
XPNPEP1	1.198400442	0.022202204
MRGPRX2	1.833203424	0.022521613
SRF	1.704102546	0.022616169
C21orf104	1.361416268	0.022712716
UFC1	1.190419123	0.02279068
IL8	2.091875372	0.022934109

IGF2	1.162776863	0.022963392
IGFLR1	1.120979398	0.023157438
CHRM1	1.011063067	0.023418141
DPCP	1.298431454	0.02349989
RAB13	1.192604885	0.024255582
PLD6	1.1654828	0.02428127
G0S2	3.339605412	0.024285499
HOXA11	1.264210168	0.024490303
NR4A2	1.034820269	0.024770885
C17orf42	1.25136634	0.025094339
BTG2	1.099529782	0.026649607
SCOC	1.220968318	0.02671287
ZNF177	1.613243686	0.027257083
LINC00239	1.254153999	0.027359176
COL3A1	1.123946279	0.027874656
MIR100HG	1.426883075	0.028214108
PHLDA3	1.283635433	0.028437649
SPATA2	1.072557074	0.028898061
FAM9B1	2.096740808	0.029268205
NEUROD4	1.205644066	0.029278538
ROBO3	1.084343079	0.029528334
GNGT2	1.253622017	0.029740227
CGF	1.561863681	0.030005297
LMLN	1.845029556	0.030707839
SRPK3	1.020249776	0.030775402
LINC00347	1.148792967	0.031127344
DCAF6	1.234538289	0.03136782
RILP	1.018406938	0.03158144
FAM6	2.014433516	0.03178432
DHRS7	1.02718241	0.031818646
LRRN1	1.513676403	0.031921276
LOC199897	1.608168567	0.032191355
SPANXN5	1.120106127	0.032523619
PIM1	1.38919577	0.033490341
SNCA	1.61282774	0.033531304
LDOC1	1.246663269	0.033727908
DEFA3	1.04275681	0.033767884
KLRAP1	1.395552223	0.033868775
NFKBIZ	1.107230207	0.033977065
HOXD2	1.365379891	0.034340678
ANP32D	1.020983322	0.034348489
LEPRE1	1.635630837	0.034525215
MAST3	1.003775985	0.035233688
STEAP4	1.08202636	0.035343698
MRPS10	1.07664626	0.035709246
NDUFV2	1.02985295	0.036507451
CHST9	1.170185678	0.036564601
SRRD	1.110414736	0.0368991
STK38	1.080464857	0.036965381
KLHDC8A	1.111434454	0.036979222
ILBF	1.031984827	0.037088599
TADA2B	1.298711023	0.03797484
DACT1	1.439521368	0.038445915
TLK1	1.076468952	0.038573267
C11orf88	1.358793393	0.038620034
ADAM20	1.100649444	0.039088945
PRUNE	1.285857797	0.039262315
NOC2L	1.180392745	0.03930318

PRSS36	1.036787047	0.039780671
RNASE4	1.099768043	0.040325548
PRF1	1.079146261	0.040586957
ALDH1A3	1.067987171	0.04058755
FBXO9	1.095457368	0.040604779
SOCS3	1.332735027	0.04079073
NEK6	1.136305851	0.04090714
C1orf145	1.445431102	0.04126177
TCF19	1.196181901	0.041474475
PIGL	1.179285848	0.041476058
TBX19	3.138882541	0.041707947
ASCC2	1.160226168	0.041744226
H19	1.04684409	0.042303601
LEKR1	1.019162375	0.042438414
EGR4	1.024844605	0.043052319
YEATS4	1.018099614	0.043152725
C22orf30	2.024825089	0.043397214
NSUN3	1.19941204	0.043813002
MTERF	1.194660884	0.044385186
LOC285456	1.610726186	0.044441471
DNAJA4	1.094355941	0.044906625
SSR2	1.195365057	0.044950596
MRC2	1.125449894	0.04517279
ARNT	1.228357477	0.045505275
RNF41	1.075818286	0.045915112
PDE6H	1.279101281	0.046634599
ADIPOR1	1.515090446	0.047530045
PPP4R1L	1.110243082	0.047671509
HCFC2	1.087093911	0.047977124
CGN	1.150079718	0.048347641
USP28	1.178062797	0.048373617
CEPB	1.178163209	0.048580083
KLC3	1.013702256	0.04924037
ALAS2	1.10362723	0.049771325
CSRP2	-4.294392087	4.34E-09
IL28	-2.547911362	0.00000718
C2orf27	-2.343688965	0.00000871
SCN4B	-1.871816089	0.0000139
FAM64A	-2.124589567	0.0000224
FAM95B1	-1.776337563	0.0000251
HOXA2	-2.431652456	0.000027
ENTPD3	-1.934303252	0.0000312
LOC652276	-1.718142651	0.0000337
CERCAM	-1.778978881	0.0000347
FAM48B1	-1.620002641	0.0000447
MUC5B	-2.481493404	0.0000481
A1CF	-1.704692731	0.000062
ASPRV1	-3.439277843	0.0000655
GJC3	-2.448756366	0.0000755
SLC22A13	-2.534094378	0.0000758
WFDC5	-2.042521599	0.000103582
CCL5	-1.624349867	0.000108594
SIM1	-1.691391911	0.000119327
LOC100129785	-2.533683823	0.000121187
PAR4	-2.508026974	0.000124424
PRKD1	-1.635652074	0.000132449
FAM163B	-1.700318734	0.000132535
TMEM235	-1.623811421	0.000159417

FLJ43903	-3.607422437	0.000203405
LHX1	-3.423309326	0.000217002
C17orf74	-4.158452241	0.000224061
EYA2	-1.428408047	0.000226888
HMCN1	-1.625293986	0.000228608
LINC00301	-1.699405923	0.000235287
FOXQ1	-1.583090492	0.000254667
PLEKHH3	-2.061941978	0.000257981
MS4A4E	-1.916592274	0.000267083
MIR17HG	-1.548819026	0.00027881
PDLIM4	-1.393117579	0.000286473
CD300LG	-2.266347988	0.000291826
B4GALNT1	-1.552735739	0.000320008
TMEM174	-2.085472808	0.000327273
GPR98	-1.500894666	0.000331517
FLJ25917	-1.860131698	0.000332558
ZCCHC23	-1.870161417	0.000344348
C14orf41	-1.699715596	0.000348674
SELV	-1.419471036	0.000357735
GOLGA6A	-1.347938057	0.000375214
C2orf16	-3.86379256	0.000384317
SLC22A24	-1.373405383	0.000403228
LOC200726	-2.072769585	0.000403311
NPTX1	-2.285296825	0.000425491
GDF10	-1.44985278	0.000435064
STXBP1	-2.808256496	0.000436838
IQCF2	-1.29621118	0.000438806
LOC100652742	-1.653071724	0.000442292
CREB3L3	-2.045282772	0.000444467
SERPINA4	-1.265393953	0.0004454
COL4A1	-1.856048445	0.000446919
LOC100129213	-1.701847205	0.000453702
FGF3	-3.336012938	0.000454205
TCF15	-2.379149813	0.0004635
DGCR9	-1.314015224	0.000468552
C17orf82	-1.322334562	0.0004706
ADD3	-1.641020235	0.000474916
VSTM2L	-2.289098084	0.000475697
LOC283856	-1.442485979	0.00047836
OR1F1	-2.180959559	0.000485684
LOC390705	-1.853617142	0.000506243
CCDC64B	-2.504569742	0.000508721
PIP	-2.43534989	0.000513613
KIRREL3-AS3	-1.732785548	0.000534309
C4orf50	-1.502157324	0.000563568
LOC100128320	-2.072156559	0.000575238
DEFB107A	-2.225454651	0.000576026
OVOS2	-2.373224567	0.000577075
DLK2	-1.137456018	0.000579034
CFL1P1	-1.750034623	0.00058306
PSG10P	-2.079453783	0.000587933
LRP2	-2.969825473	0.000594497
LOC728671	-1.860295704	0.000604731
C2orf61	-1.657057298	0.000611752
MAST4	-1.274290695	0.00062205
HTR6	-1.67780665	0.000622194
ZNF16	-1.794770789	0.000623929
ADAMTS16	-1.159578264	0.000653133

LOC729080	-1.508222737	0.000670785
MIER1	-1.387901018	0.000684553
ZNF556	-1.616604969	0.000692257
DPCR1	-2.217471338	0.000701495
FGL1	-1.698970436	0.000708776
OR4C6	-3.211448914	0.000720705
ZDHHC8P1	-1.64847419	0.000732336
DPYSL3	-2.260628013	0.000747659
LANCL3	-1.529418472	0.000751187
GRM4	-1.590275009	0.000768923
TMEM150A	-2.196602742	0.000770947
LOC285103	-1.377637666	0.000786638
CLIP2	-1.081590421	0.00081644
SCGB1D2	-1.148069504	0.000830754
OTUD7A	-1.349543278	0.000846028
TRH	-3.10881331	0.000853623
LOC100132474	-1.532567778	0.000869713
C7	-1.807323836	0.000879324
RASD1	-1.667664303	0.000880262
ND4L	-1.707193825	0.00088296
FAM48B2	-2.177755861	0.000897418
FZD9	-2.253199296	0.000912927
TBX6	-2.091521555	0.000947518
GABRE	-1.801090358	0.000955287
LOC100129048	-1.094543559	0.000964429
CYP11B1	-1.635750466	0.000992909
LOC100652922	-1.9436937	0.000994131
OR10R3P	-1.118049271	0.001015213
LOC283270	-1.993976165	0.001027851
GPT	-2.881176979	0.001037427
PTK6	-1.499704083	0.001043097
HOXA6	-1.419990855	0.001049093
RPL39	-1.191002897	0.001054054
MEP1A	-1.635129655	0.001056451
CCL20	-1.990499021	0.001071841
PODNL1	-1.488894687	0.001073147
SLC2A12	-1.410238275	0.001081502
RBP5	-1.590745382	0.001093696
PLEKHN1	-1.785753173	0.00111481
SOX21	-1.21164401	0.001119009
GINS2	-2.437038302	0.001125305
LOC100129148	-1.429795595	0.001128194
FADS6	-2.102118671	0.001131235
SNAR-C3	-2.320194714	0.00116078
NEUROG1	-1.284552832	0.001161247
KRTAP19-1	-2.231581327	0.001172936
LOC441204	-1.599621499	0.001175067
LOC100130954	-2.114173274	0.00117947
DYNC2LI1	-1.297690999	0.001193268
MYOD1	-2.285421322	0.001210564
FLJ30403	-1.697468324	0.00122654
GAFA1	-1.05405986	0.001238208
AACSP1	-2.374155163	0.001248457
NAG20	-1.525398069	0.001249014
NKAPP1	-2.929541412	0.001249489
LOC100130800	-1.7662254	0.001257031
FAM19A4	-2.713044144	0.001258055
GJD4	-1.370524316	0.001281919

L2HGDH	-1.494509871	0.001284121
OR5I1	-1.371702054	0.001315081
LINC00302	-1.479205965	0.00132847
ADH4	-1.813180145	0.001367703
OR7D4	-1.347854869	0.001370115
LOC399939	-1.650711413	0.001401673
POM121L8P	-2.218789727	0.001407141
TCEB3C	-2.383951982	0.00140938
OR2L3	-1.416828618	0.001413886
ACTH1	-1.350216256	0.001417755
INHBA	-1.724782836	0.001418529
BTG1	-2.528701226	0.001422216
LOC646498	-1.500512828	0.001423936
PTGER1	-1.726838497	0.001428524
RDM1	-1.349833781	0.001437063
SOST	-1.271190704	0.001437547
DGCR10	-1.633877135	0.001439085
PDILT	-1.537003381	0.001443331
LOC647983	-1.708968286	0.001446911
OR10A6	-1.215521754	0.001468445
CDH4	-1.853811487	0.001532025
BCL2L10	-1.691684222	0.00154752
LOC389199	-1.872836959	0.001595546
SGK196	-3.215728466	0.001599077
C1orf138	-1.682025293	0.00164114
CENPO	-1.149039305	0.001657416
OR2B9P	-1.48507068	0.001670172
LOC116437	-1.16764713	0.001681989
PCBP3-OT1	-1.721356671	0.001683647
LUZP4	-1.152752359	0.001693053
SULT4A1	-1.222101601	0.001697502
ASTL	-1.747278831	0.001724603
GSG1	-1.112053632	0.001756413
MMP14	-3.026922763	0.001757786
CEP72	-1.262336053	0.001769004
LOC100131170	-1.595183479	0.001771581
DKK3	-1.132045866	0.001794253
UGT1A6	-1.130139534	0.001829009
FAM106A	-1.369013876	0.001852586
SNORA80	-1.614036671	0.001870348
CHKA	-1.307609305	0.001874273
TMEM132E	-1.404768396	0.001889343
LOC100130278	-1.254406864	0.00189461
FAM5C	-1.512943067	0.001900069
LOC728208	-1.207234335	0.001903248
HSPA12B	-1.963385249	0.001935314
NODAL	-1.275762701	0.0019382
OSTBETA	-1.156324393	0.00196788
C11orf52	-1.358643001	0.001972544
LOC100134368	-1.256990609	0.00199452
ANKRD20A4	-1.71042129	0.002000701
PREX2	-1.024643223	0.002004513
GRHL3	-1.206518906	0.002011986
AVPR1B	-1.353419947	0.00201224
SHANK3	-2.398613091	0.002025675
SEMA6C	-1.777723499	0.00203534
HYAL1	-2.679178979	0.002041051
PRAMEF4	-1.682554971	0.002051607

MED1	-1.233235367	0.002054137
ARVP6125	-2.002905227	0.002061783
PLA2G4F	-1.177957251	0.002063425
LINC00467	-1.149132094	0.002076848
SYNDIG1L	-1.761346774	0.002109285
GPRIN1	-1.15221167	0.002109506
XAGE1A	-1.344866411	0.002135955
ZP4	-1.231437525	0.002139165
LOC100128653	-1.222848609	0.002152956
MAN1B1	-1.788671388	0.002160587
FAM41C	-1.394091994	0.002163963
OR4N4	-1.544409268	0.002175455
GPD1	-2.657261719	0.002187045
KCNB2	-1.122733418	0.002222069
MAGIX	-1.035794171	0.002224269
FAM71F1	-1.562304705	0.002290875
DCDC1	-1.274160668	0.002295617
EGFL6	-1.248469607	0.002317378
SERPINB7	-1.236631173	0.002326982
OR51L1	-1.340899805	0.002329443
IFITM5	-1.760872732	0.00234087
CA7	-1.111636092	0.002344986
PTPRH	-1.357684772	0.002364705
RNU105C	-2.498579893	0.002370776
FLJ42022	-1.100190923	0.002378505
THNSL2	-1.058738516	0.002388154
LOC100130354	-1.728244414	0.002447109
MEF8	-1.182828991	0.002462827
PRSS35	-1.488088808	0.002503991
GOLGA6L10	-2.220512371	0.002519638
SIGLEC6	-1.763399107	0.002542045
RBFOX1	-2.599807669	0.002545287
IL24	-2.113701922	0.002591733
GGT6	-2.02045084	0.002638861
MYLK2	-1.427249485	0.002655954
SHOX	-1.760631933	0.002669988
LOC100129775	-1.363777324	0.002696778
RNASE7	-1.489968621	0.002705564
CREG2	-1.457101689	0.002708432
FAM201B	-1.556748975	0.002717767
FGF17	-1.674360624	0.002722211
LOC642776	-1.645581157	0.002726617
TSPAN16	-1.77599481	0.002727973
ENTPD2	-1.801753584	0.002730923
TERT	-1.558841985	0.00273773
RNASE12	-1.211313172	0.002775656
LOC145837	-1.293465952	0.002780595
OR52E2	-3.030169109	0.002783758
LOC100507505	-2.238771959	0.002825973
LOC729770	-1.922003841	0.002840872
KCNK13	-1.572317963	0.002856255
GABRR1	-1.458373076	0.002861694
RAB36	-1.639200595	0.002875625
OR2M5	-1.745273337	0.002895441
LINC00273	-2.667112565	0.002925871
DUX3	-2.225371564	0.002933861
C22FG	-1.424893459	0.00297525
HOXB6	-1.581381839	0.002988278

ULBP2	-1.250008158	0.002991136
RUSC1-AS1	-1.479730164	0.00299445
TSPY26P	-1.167066397	0.003061183
TMEM52	-1.261494706	0.003069132
LINC00371	-1.136886443	0.003078858
SLC35G5	-1.242366427	0.003082054
LINC00112	-1.604043794	0.003100721
RUFY4	-1.408915907	0.003102892
MMP1	-1.546089268	0.00311321
LOC100128811	-1.175393585	0.003130993
PDE8B	-1.623544121	0.003132427
APOC4	-1.035408115	0.003144191
LOC283140	-1.269651338	0.003158286
LOC100653108	-1.505049521	0.003169666
LINC00111	-2.283517985	0.003185398
ERPI	-2.885808003	0.003191104
SPACA1	-1.288724136	0.003207257
SLC17A6	-1.617644466	0.003239144
PROX2	-1.388520504	0.003265494
CLCN2	-1.310503604	0.003274063
NNMT	-3.245357325	0.003276317
LGALS14	-1.0580696	0.0032764
LOC100653245	-1.991313241	0.003282634
C8orf74	-1.423029348	0.003290572
DNAJB3	-1.551793653	0.003293998
LOC284242	-1.70312731	0.003305672
SLC45A2	-1.308572962	0.003317608
SNORA60	-3.997239982	0.003330065
ST8SIA3	-1.191022594	0.003340896
RNF150	-1.69691743	0.003343037
ZNF662	-1.425009585	0.003402124
FAM83E	-1.167365518	0.003406384
CTSL3	-1.246918388	0.003408962
NACAD	-1.163471189	0.003444987
IGJ	-2.788314361	0.003460413
KCNN3	-1.453878054	0.003466305
SH3GL1P3	-1.237212387	0.003475245
BGN	-2.014090294	0.003488457
PCBD1	-1.416355418	0.003494844
IL36B	-2.582544632	0.003496117
C17orf47	-1.548945411	0.003519906
PSG8	-1.15697575	0.00352112
SCAMP5	-1.233791794	0.003523859
C20orf85	-1.069783916	0.003553873
MIA	-1.388469416	0.003562675
KNCN	-1.431425553	0.003577843
OR51G1	-1.114691991	0.003589767
C5orf64	-1.542667255	0.003608267
SPANXA1	-1.221282137	0.00360937
HIGD1B	-1.491009695	0.003609407
MPV17L	-1.246168475	0.003662487
LOC728613	-1.891106544	0.003687645
AOC4	-1.490361675	0.003729775
LOC100129393	-1.402712581	0.003744724
TRIM49	-1.07560959	0.00374817
RGL1	-2.465130439	0.003748905
KIAA1984	-1.264738085	0.003749137
LOC100132686	-1.129784632	0.003749345

C1orf98	-1.675049096	0.003768105
RAB19	-1.037734272	0.003787819
SERTM1	-1.615393961	0.003796799
PPP1CA	-1.002796368	0.003830487
FAM123C	-1.059077488	0.003840435
PEX11G	-1.50792612	0.003854879
TMEM54	-1.216408572	0.003882996
F7	-2.564966685	0.00388534
ZNRD1-AS1	-1.340361059	0.003890498
LOC284561	-1.146977184	0.003926886
ZNF833P	-1.291271474	0.003965806
LOC644554	-1.91214335	0.003978076
SGSM1	-1.490809326	0.003987625
HEATR4	-1.07795397	0.003992428
LOC653712	-1.779423135	0.004007642
IL36A	-1.018293266	0.004016667
LILRP2	-1.223008524	0.004023715
OR5AS1	-1.955907309	0.004028593
OR4F29	-1.082925297	0.004037903
TTTY5	-1.365072774	0.004058098
TRIM17	-1.061595155	0.004065895
CSAG1	-1.835524984	0.004090659
LOC100128922	-1.478070439	0.004147948
LOC100131581	-1.421980609	0.00416921
LOC100133091	-1.440858546	0.0041978
MDFI	-2.31442298	0.004239505
FAM181B	-1.147818999	0.004295907
MAT1A	-1.25963941	0.004316705
TBC1D28	-1.165955677	0.004338906
SSX4B	-1.676926111	0.00434219
LINC00469	-1.309282838	0.004355508
IL26	-2.034679725	0.004389123
HSPB3	-1.132293372	0.004397517
CCL19	-1.286868528	0.00441014
RND157	-1.018450314	0.00444341
SERPINB13	-2.336271122	0.004470791
LOC100128851	-1.260747006	0.004474595
FBXO27	-2.180098891	0.004478036
ITGBL1	-2.45533908	0.00448978
DYNC1I1	-1.324660883	0.004492154
LOC642620	-1.174723091	0.0045058
LOC147670	-1.741496377	0.004523366
LAMA4	-1.169651113	0.004526615
LOC100130865	-1.138576746	0.004540701
LOC100131195	-1.812060239	0.004561491
OVOL2	-1.315338433	0.004569906
CD209	-1.175368308	0.004574039
EML5	-1.181143118	0.004590607
OR2T27	-2.57773869	0.004617623
IMP5	-1.241937125	0.004646016
FUT5	-1.764771253	0.004689488
GPM6B	-1.124964656	0.004730601
C16orf3	-1.822932761	0.004750564
PHYHIP	-1.394253601	0.004762213
LOC400743	-1.453523121	0.004777895
ONECUT3	-1.397463615	0.004780106
PLP1	-2.507064435	0.004810828
CENPT	-1.256617377	0.004884771

CT45A5	-1.941977047	0.004893664
LOC100134409	-3.473536097	0.004931233
HLA-E	-1.510566026	0.004936958
LOC392364	-1.000711522	0.004937559
CLP2	-1.137602423	0.004956165
LOC100131894	-1.453515077	0.004992732
LOC284219	-1.964360757	0.004997284
EFHC2	-1.329866809	0.00511007
GOLGA8F	-1.398856342	0.00511518
TRPC3	-1.928772392	0.00512643
C15orf59	-1.496319847	0.005143494
LOC283731	-1.271100547	0.005157267
UGT2B11	-1.787143227	0.005181361
RNF151	-2.261200993	0.005222089
LOC285300	-1.163779457	0.005256282
LOC253044	-1.910444702	0.005270076
MAP3K10	-1.202778608	0.005312223
LOC100128184	-1.381531394	0.005328747
LOC729668	-1.367304514	0.005331162
LINC00410	-1.326930507	0.005385016
NDP	-1.013714838	0.00539154
VAX2	-2.15698335	0.005394426
FLJ44124	-2.515667234	0.005420219
LOC729444	-1.855119679	0.005447238
ATP4B	-1.271998962	0.005455482
DNM1	-1.005950143	0.005472038
GSTTP2	-1.757316569	0.005481553
FAM166A	-1.3824816	0.005490094
WTIP	-1.35682172	0.005491648
LOC100270679	-1.273695843	0.005495479
LOC100130442	-1.617795489	0.005545616
FAM132B	-1.219310045	0.005547701
MDH1B	-1.185202651	0.005552072
TTTY22	-2.14327411	0.0055621
TNC	-1.262438068	0.00558717
LOC63930	-1.372198793	0.005623528
PRSS27	-1.553398738	0.005651198
HSP90AB1	-1.271909963	0.005685193
LOC100288884	-1.165956696	0.005706483
DAND5	-2.475160703	0.005714322
C8orf66	-1.664488572	0.005716498
TEF	-1.422106323	0.005757679
ZNF682	-1.186117967	0.005766886
GOLGA6L6	-1.117544283	0.00589393
FLJ21408	-1.463659927	0.005899154
RARRES2	-1.592735768	0.005916836
IGLON5	-1.486553854	0.005923257
ITIH3	-1.624798195	0.005931391
IL32	-1.774349757	0.005933782
CD97	-1.468818661	0.005955923
LOC100128333	-1.138126161	0.005978021
EMR4P	-1.070289802	0.005981879
C1QTNF2	-1.262360146	0.006004564
LOC143286	-1.467035598	0.006026517
FAM180B	-1.170785468	0.006114839
IGSF11	-1.596092864	0.006120176
DDX24	-1.128435511	0.006148394
ZC3H10	-1.039430782	0.006166738

LYZL1	-1.914310939	0.00619518
CRMP1	-1.63161877	0.006204421
RFPL1-AS1	-1.178261277	0.006220948
STH	-1.490492209	0.006235666
PRKD2	-1.428985875	0.006255343
PGF	-1.252995322	0.00631551
ANKMY2	-1.135734161	0.006336033
C1QTNF1	-1.344052077	0.006339132
SNAR-C4	-1.899621026	0.006341431
KRTAP10-5	-1.248425718	0.006347902
REG3A	-1.448383025	0.006378407
OR7E37P	-1.020661221	0.006403938
SPDYE8P	-2.282648811	0.006424355
PRAMEF15	-1.613675578	0.006425477
C9orf79	-1.241592488	0.006426869
SH2D4B	-1.019665033	0.006431586
ADSSL1	-1.027593073	0.00643642
CASR	-1.113147814	0.006444779
TAF1L	-1.852960474	0.006455285
SH3D19	-2.879598822	0.006505898
CCDC135	-1.495389883	0.006522822
LOC100128751	-1.158614725	0.006541534
UBD	-2.040618932	0.006576643
ZC3H12D	-1.402028791	0.006581277
LSP1	-1.69869343	0.006587346
CRHR1	-1.087232312	0.00660904
OR56A4	-1.474935352	0.006640183
LOC389831	-1.591358743	0.006708371
LOC100129316	-1.028324738	0.006724355
OR4F6	-1.092408655	0.006773978
RDH13	-1.020309628	0.006853222
HMGB3P1	-1.460745751	0.006875941
TRDN	-1.11586428	0.006879853
HOXD12	-1.703600062	0.006910762
LOC202181	-2.046872794	0.006943741
MYOZ3	-1.176537103	0.00697616
LOC100128386	-1.504760125	0.006997806
GCM2	-1.815352115	0.007009468
LRSAM1	-1.331746657	0.00704779
KIAA1755	-1.015428774	0.007079755
PROM2	-1.471349049	0.00709961
LOC100127904	-1.907592515	0.007139427
LINC00242	-1.706006017	0.007161137
LOC100129125	-1.60330386	0.007215071
LOC100128531	-1.117046608	0.007258984
OVOS	-1.096566227	0.007303072
C1orf95	-1.091309706	0.007312981
MS4A15	-1.023959386	0.007329346
CDH24	-2.285927942	0.007361114
POLQ	-1.215537751	0.007434881
ZNF704	-1.156800852	0.007475131
C20orf166-AS1	-1.323467993	0.007554554
DCDC2B	-1.587274252	0.007557262
HCFL	-1.516340818	0.007638208
ARPM1	-1.109615413	0.007662637
PRKACG	-1.189762122	0.007671711
LOC100130849	-1.213638425	0.007676795
MYL3	-1.225020952	0.007678644

TAS2R14	-1.222404998	0.007714502
C6orf191	-1.020379604	0.007720819
C21orf106	-1.268545306	0.007770086
GATA4	-1.060458521	0.007857126
EIF4E1B	-1.235509441	0.0078694
RGMA	-1.059280668	0.007881261
ANKRD30BL	-1.350631348	0.007896484
TRPC6	-1.020777718	0.007934121
ZP3	-1.242993504	0.007956969
TECRL	-1.670642631	0.008020155
LOC100128366	-1.053151936	0.008023051
C4orf11	-2.199004866	0.008133287
KRT27	-1.18497003	0.008196769
RTKN	-1.077412529	0.008216476
ANKRD20A5P	-1.262537492	0.008287323
LOC285758	-2.27641311	0.008304351
SCARNA5	-1.397718996	0.008311421
HAPLN2	-1.532018756	0.008315484
LOC100132526	-1.91972439	0.0083158
LOC100130193	-1.517217151	0.008342819
CLSTN3	-1.124490801	0.008352545
LOC284669	-1.713497662	0.008359958
MYLPF	-1.185442949	0.00839458
LOC100131043	-1.062031312	0.008468518
LOC727677	-1.129837505	0.008504291
PTGR2	-2.35481725	0.008522075
SH3GL1P2	-1.019453806	0.00853387
AZFP	-1.047981178	0.008567606
SLC24A5	-1.943056685	0.008573034
C1orf61	-1.118406754	0.008584761
C10orf62	-2.108257368	0.008630826
PRSS47	-1.052438763	0.008662142
OR2L2	-1.638726085	0.008721373
BMP15	-1.067961085	0.008870814
GKN1	-1.282779385	0.008879094
CFL1	-1.032614046	0.00899371
LOC100287879	-1.083393555	0.009040703
AANAT	-1.6180827	0.009043204
FBXO44	-1.104247498	0.009071975
TAS2R30	-1.117106202	0.00908092
FAM196A	-1.126733977	0.009162029
FLJ34223	-1.271677483	0.009212958
MD5R	-1.152135928	0.009262016
ZSWIM5	-1.255454088	0.009303248
IL21	-1.109544249	0.009321355
SPTBN5	-1.056055417	0.009401826
C17orf72	-1.443724095	0.009436946
LOC442122	-1.383405346	0.009478132
S100A5	-1.545612386	0.009508803
OPN1MW	-1.277749014	0.009526812
LOC100272216	-1.663820455	0.009566326
FYN	-1.220734444	0.009591905
ANKRD63	-1.044932993	0.009592602
C7orf54	-1.217559126	0.009599442
FLJ37786	-1.01716516	0.009601999
LGALS7	-2.255028714	0.00965887
IL1RAPL1	-1.534695361	0.009683816
C7orf61	-1.537186826	0.009722774

RP9P	-1.857398805	0.009800778
KRTAP20-1	-1.827565451	0.009844782
SYNGAP1	-1.048971427	0.009855269
LGALS2	-1.004677433	0.009861612
ANKRD18A	-1.141326057	0.009886122
FOXD4	-1.142393904	0.009962815
MPP2	-1.18583615	0.009974847
DNM1P46	-1.599210412	0.009981477
FILIP1	-1.057346385	0.010015944
LOC100130480	-2.173215914	0.010044951
FLJ41327	-1.250161073	0.01004998
LOC100130579	-1.374752019	0.010102334
ASIP	-1.24061176	0.010137437
ATP4A	-1.110747253	0.010173752
OR2A5	-1.574661074	0.010212768
MAML3	-1.009692037	0.010278528
RAX2	-1.326571348	0.01029109
COTL1	-1.605115515	0.010346803
FLJ13224	-1.006064237	0.010362206
TAS2R60	-1.882523755	0.010366991
SLC28A1	-1.060019611	0.010411701
GDI2	-1.028693583	0.010436926
C1orf140	-1.875663054	0.010482852
PAX5	-1.088182377	0.010503396
UPK3A	-1.482255404	0.010529118
LOC388882	-1.240506664	0.010621768
POM121L1P	-1.112430002	0.010644728
LOC399829	-1.326270176	0.010650993
ST3GAL4	-1.180463266	0.010656789
OR7E5P	-1.240517109	0.010658801
CAND1	-1.044696427	0.010706736
FAM92A3	-1.68861301	0.010766207
GUCA2B	-1.058979475	0.01078349
KCNV2	-1.219709753	0.010861182
ZNF667	-1.115774092	0.01087901
SIX3	-1.425898317	0.010889315
KCNE4	-1.66600411	0.010912413
IGFALS	-1.013234023	0.010918662
LOC100233156	-1.141513482	0.010961105
AADACL3	-1.131437333	0.010977385
KIAA1161	-1.039841779	0.010980291
OR2AT4	-1.168513051	0.010985308
DEFB132	-1.180229776	0.010990156
LOC729706	-1.941396197	0.011031006
PRSS16	-1.49861727	0.011127141
LCE2C	-1.091654084	0.011129105
PIH1D2	-1.999944158	0.011136767
LOC643669	-1.283368963	0.011151572
GTF2IRD1	-1.17652301	0.011179711
MGC23270	-1.033333395	0.01134846
LOC284412	-1.023854156	0.011376873
ARHGDIB	-1.032454119	0.011382543
SEZ6L	-1.072905673	0.011397187
KRTAP5-3	-1.095923728	0.011472691
KCNH6	-1.417127866	0.011476171
PRO0471	-1.791790708	0.011508994
GGNBP1	-1.004324204	0.011571954
NADKD1	-1.520727358	0.011640927

HOXB13	-1.120515347	0.011662073
ZNF503-AS2	-1.395436218	0.011689056
OR51V1	-1.305876397	0.011733635
TSPAN15	-1.195700731	0.011758305
LOC100131742	-1.059783671	0.011769676
C2orf66	-1.085402118	0.01180386
LOC100133131	-1.214300506	0.011825809
LOC283738	-1.01948299	0.011825947
CARD10	-1.085005689	0.011866791
LOC730139	-1.821958348	0.011891547
FLJ16126	-1.046813914	0.011899825
PPP4C	-1.334157228	0.011905593
PLAC4	-1.188669443	0.011932111
LRRC19	-1.231629947	0.011980146
DMBT1	-1.207654424	0.012047959
S100A14	-1.412417511	0.012077374
OR51D1	-1.051757142	0.012086352
MAFIP	-1.963917495	0.01227558
SLC12A8	-1.428158593	0.01237302
MCART6	-1.073605183	0.012390769
WFIKKN2	-1.258087585	0.012408622
MAGEC3	-1.858045	0.012531331
LOC729626	-1.477213497	0.012656616
CHRNG	-1.268874001	0.012678682
ARHGAP39	-1.160095413	0.012704836
SSC5D	-1.257404342	0.012738245
CES3	-1.275136089	0.012743915
SPRR2E	-1.037560118	0.012777087
LOC100128640	-1.099587601	0.012926577
MMP2	-1.463151796	0.01294583
C16orf73	-3.044670898	0.01297384
LOC257152	-1.584900635	0.012984111
UPK1A	-1.436147783	0.013018304
VWA5B2	-1.022476602	0.013060734
ASS1	-1.148290556	0.013121147
LOC100130741	-1.184734821	0.013149862
SLC4A11	-1.247913077	0.013212648
FOXN4	-1.012896205	0.013288183
KRT18P55	-1.099869394	0.013329668
LOC100130285	-1.213091887	0.013431441
CHRNA2	-1.391634166	0.013442922
DHRS2	-1.310248224	0.01344924
HIST2H3A	-1.670581801	0.01346821
TSIX	-1.17631171	0.01348761
LOC146795	-2.007671507	0.013514257
LOC100144602	-1.237839624	0.013591988
KCNQ2	-1.424315414	0.013682783
C17orf105	-1.035797261	0.013704045
XRCC2	-1.429734473	0.013714478
KRTAP5-8	-1.191847245	0.013873972
TNFRSF19	-1.327132596	0.013952158
TXNDC2	-1.484861678	0.013967412
C8orf71	-1.008867519	0.013975204
SPINK14	-1.34415601	0.014005813
C18orf15	-1.302112949	0.014119124
LOC100128977	-2.019980151	0.014297871
ENTPD8	-1.264010627	0.014623346
CRYM-AS1	-1.193635879	0.014636694

LIPH	-1.060933132	0.014728999
IPO13	-1.295188908	0.0147442
RPA4	-2.078652066	0.014748104
CELF6	-1.03033649	0.014791611
RND1	-1.003013523	0.014792738
SH3BP5L	-1.037381891	0.014821554
FLJ33065	-1.137801027	0.014824206
TDGF1	-2.665932634	0.014829994
LOC100130175	-1.196385004	0.014841485
SLC30A3	-1.109205334	0.015001488
LOC400752	-1.035728142	0.015042813
LINC00479	-1.46249376	0.015088296
ABO	-1.955963972	0.015117454
FAM101A	-1.480379542	0.015171082
PRSS8	-1.578294107	0.015186186
LOC653061	-1.920593728	0.015251674
LGI4	-2.605211297	0.015314215
GLYAT	-1.405386546	0.015351763
LOC650293	-1.643087833	0.015403121
LOC100128501	-1.061038009	0.015426388
HIST1H2APS1	-1.150068689	0.015473996
LOC100132197	-1.635927407	0.015582975
LRRC3C	-1.115716308	0.015602693
VHLL	-1.114918749	0.015622012
PGAM5	-1.097218918	0.01566971
FZD4	-1.182636077	0.015820854
OR10G2	-1.099708541	0.01586964
GGT8P	-1.518247841	0.015890666
MUC13	-2.405049667	0.015913424
KLK8	-1.141527355	0.01594529
PHKG1	-1.385224853	0.016018587
TSGA10IP	-1.936193163	0.016049086
KRTAP12-2	-1.015165719	0.016226961
CCDC116	-1.036283042	0.016245511
CALHM3	-1.315221939	0.016283147
FAM90A7	-2.066381638	0.016428165
OR9H1P	-2.629896776	0.016436223
LOC100289650	-1.338895216	0.016454731
LOC440742	-1.285555227	0.016464873
HTATSF1P2	-1.293734554	0.016576377
LINGO2	-1.646205091	0.016656956
RPL23AP64	-1.197295038	0.016820113
CACHD1	-2.511532815	0.01689968
PRAMEF2	-1.133847361	0.016908901
OR2C1	-1.253108639	0.017076619
ART4	-1.257530674	0.01717333
SCN4A	-1.019416991	0.017179823
CDRT8	-1.311920679	0.017253384
HORMAD2	-1.205379632	0.017332917
KRTAP4-5	-1.049495105	0.017509069
RGPD6	-1.932927608	0.017577545
DKFZp451A211	-1.067490142	0.017590862
LOC100132014	-1.269144693	0.017702438
SUSD2	-1.094122421	0.017759626
LOC100287314	-1.368558801	0.017763581
GOLGA6L1	-1.284565339	0.01783494
LOC284950	-1.230777357	0.017860577
NKPD1	-1.399998348	0.017873904

CHP2	-1.019372299	0.017896503
CRX	-1.300468043	0.017981221
LOC100133319	-1.014081513	0.01811334
CIDEA	-1.434863907	0.018113601
LOC100499221	-1.43209893	0.018122568
PLA2G2F	-1.14340584	0.018213124
DNAJC5B	-1.351204504	0.018282268
GOLGA7B	-1.087824148	0.018303032
LOC100130542	-1.023930693	0.018340397
SLC9A4	-1.73840762	0.018378256
LOC100128001	-1.515895851	0.018395579
SCXA	-1.064838879	0.018415366
HOXB5	-1.275131216	0.01845774
OR2T6	-1.045284094	0.01852569
NANOG	-1.220075451	0.018626133
TTC9B	-1.570509091	0.018738477
OR2M7	-1.305372589	0.018745951
FLJ45079	-1.10747855	0.018761153
GAMT	-1.096678144	0.018862576
PGPEP1L	-1.279722055	0.018892327
SLC22A25	-1.015844531	0.018900328
FGF14-IT1	-1.242579531	0.018943527
ZDHHC22	-1.512943918	0.019030181
LOC100130453	-1.123208413	0.019247348
OR2V2	-1.127423422	0.019267126
RDH8	-1.084951791	0.019291936
PRINS	-1.53351848	0.019301216
LOC340515	-1.641943738	0.019378252
ARTN	-1.050008621	0.019494415
SULT6B1	-2.183584448	0.019552539
MST1P9	-1.176232782	0.01989566
NLRP7	-1.239413333	0.019917215
ACTG1	-1.066351218	0.020008216
REXO1L1	-1.683947183	0.020045776
TUBG2	-1.350162131	0.020049155
FOXL2	-1.444288727	0.020095474
LOC158572	-1.47486158	0.020139972
LOC100506533	-1.026476366	0.020181767
LOC286154	-1.157041567	0.020323472
PCDH10	-1.413479941	0.020365892
BRSK1	-1.134384145	0.020412391
LOC144817	-1.000319136	0.020448324
SETMAR	-1.037255771	0.020462236
LOC100289079	-1.031388438	0.020463613
SFRP1	-1.18600646	0.020480361
BUB1B	-1.582456691	0.020527668
FAM25A	-1.314108701	0.020550056
SLC32A1	-1.506893656	0.020592133
LOC100128950	-1.191067156	0.020594536
MTMR10	-1.048817298	0.020785421
STK16	-1.734974156	0.020896506
LOC100128644	-1.134073675	0.020908022
GLOD5	-1.314007746	0.021110018
LOC143666	-1.509057958	0.021121537
MGC4859	-1.100191972	0.021141897
LOC338667	-3.152794077	0.021173575
LRRC14B	-1.968001035	0.021290231
DUSP26	-2.051581022	0.021382436

EXTL1	-1.279056993	0.02161604
ACBD7	-1.452915989	0.021693171
IGSF21	-1.083907966	0.021723799
FSTL3	-1.177591366	0.021996481
GPR101	-2.02700946	0.022186121
DLX4	-1.411006861	0.022205659
GDF1	-1.687777252	0.02223232
FAM27L	-1.445727255	0.022458797
GPHA2	-1.027682173	0.022484674
LOC402160	-1.150721858	0.022584664
LOC731779	-1.135963231	0.022628413
OR2Z1	-1.589505509	0.022638109
LOC147004	-1.440581895	0.022654493
FAM27A	-1.327288778	0.022664897
FAIM3	-1.139530144	0.02273643
SH3RF2	-1.577018409	0.022821591
LRIT1	-1.927475024	0.022866905
RAP1GAP	-1.105215669	0.022871667
SPDYE3	-1.525718936	0.022991459
DBX1	-1.229073911	0.023038961
CPNE9	-1.160860362	0.023059285
PTGR1	-1.723947206	0.023255755
GHDC	-1.059351739	0.023544526
FUT7	-1.048417683	0.023613099
LOC100128226	-1.222547287	0.023616374
MCC	-1.153551044	0.023684462
FLJ35424	-1.2902432	0.023873447
SOX3	-1.565844837	0.023942583
NEAT1	-1.344802758	0.024339984
CSH1	-1.046565213	0.024377621
GBX2	-1.637049297	0.024396333
KRT79	-1.199933732	0.02453818
LCE3A	-1.723204741	0.024581532
ADRA2C	-1.729221335	0.024621555
OPALIN	-1.030833387	0.024639796
LOC100132859	-1.656354715	0.024778074
MYCNOS	-1.150798661	0.024849936
CHSY3	-1.215719009	0.024879752
DMC1	-1.037919525	0.024889723
OR8H2	-1.999523108	0.02503997
CD86	-1.895145621	0.02522629
SRPX	-1.109133722	0.025311244
LINC00328	-1.363402779	0.025395339
LOC389602	-1.115982028	0.025414245
KRTAP10-4	-1.106758858	0.025420074
COL13A1	-1.125594719	0.025567772
SHANK1	-1.53605527	0.025609751
TACC2	-1.370857406	0.025879519
IL17RC	-1.097150374	0.02593628
TOB2P1	-1.284839839	0.025941707
MTSS1L	-1.523833062	0.025981725
TRIM31	-1.037976926	0.026165938
LOC399875	-1.301471598	0.026239544
UNQ9370	-1.832007975	0.026276297
BSX	-1.197706764	0.02634811
SHROOM1	-1.002235493	0.026366491
RNF165	-1.31801223	0.026431789
BRD7P3	-1.489896159	0.026558591

SLC35G1	-1.475139013	0.026709097
FLJ22763	-1.217651174	0.026866858
LOC100127885	-1.393722076	0.026873726
TNNT2	-1.282413303	0.026909015
C3orf51	-1.298532229	0.026978429
KRT8P41	-1.325358045	0.026989257
MAP4K5	-1.064833551	0.027133073
C22orf36	-1.457061776	0.02728715
P4HA2	-1.157304498	0.027375884
OLIG3	-1.256954645	0.027385311
ALDH1L1	-1.029927487	0.027553237
IGFL3	-1.498932555	0.027606841
LOC100131242	-1.160837028	0.027809078
GAGE7	-1.882259127	0.028062853
SORCS3	-1.020707131	0.028321029
BZRAP1	-1.323587054	0.02835126
MAGEA4	-1.03641033	0.028383776
ADRA1B	-1.019466651	0.028507416
KIR3DL3	-1.839486617	0.028575828
S100A3	-1.145467552	0.028606191
ABCA9	-1.216976272	0.028692604
UGT2B7	-1.252963594	0.028792364
KRTAP5-7	-1.426048728	0.029020534
LOC255654	-1.551308992	0.029326275
PEG3-AS1	-1.268962588	0.029354828
NLRP8	-1.359977572	0.029477559
SNORA73A	-1.698846165	0.029737635
TRAIP	-1.245269913	0.029967827
PRG2	-1.179981361	0.030084104
LOC728723	-1.301442563	0.030236678
CDH16	-1.819584183	0.030241783
TXNIP	-1.084928848	0.030444686
LOC339874	-1.329549335	0.030642293
FJX1	-1.294053855	0.030831324
CBLN4	-1.378709688	0.030866727
TMEM233	-1.64395114	0.031040619
FBXW10	-1.69541366	0.031044054
FAM182B	-1.422770796	0.031083053
CLSN3	-1.021508812	0.031205026
LOC401176	-1.086045294	0.031396161
LOC284440	-1.896491295	0.031500489
FOXD4L2	-1.606596105	0.03158801
LOC440040	-1.741986916	0.031749679
PLA2G4D	-1.099461822	0.031877674
UCA1	-1.01387971	0.032276312
CDHR3	-1.08495889	0.032403131
C1orf210	-1.170116193	0.032518098
ATPAF1-AS1	-1.196638755	0.032633071
KCNN1	-1.336737532	0.032717009
CCBP2	-1.392009748	0.032725126
SIX1	-1.454862656	0.032735318
BDNF-AS1	-1.221198923	0.03296723
DEFB109P1	-1.057745289	0.033201323
SDCBP2	-1.538187581	0.0332768
TTTY1	-2.072557213	0.033404663
DEFB119	-1.053985273	0.033438855
FLJ41455	-1.166749187	0.033784644
OR4P4	-1.371407945	0.033883754

PPP6R1	-1.179693089	0.033968097
HOXC11	-1.997689022	0.034007941
GNLY	-1.643423576	0.034061925
NPIP	-1.486110529	0.03408186
NRG2	-1.015113347	0.034245353
ANGPTL4	-1.393778156	0.034459222
LOC646034	-1.001889103	0.034597299
HABP2	-1.301339363	0.03461796
ZNF423	-1.980593969	0.034959041
FREM2	-1.069025536	0.034967088
MFSD6L	-1.603958994	0.035134208
DRD5	-1.656023961	0.035143048
IL2RG	-1.173313236	0.035183232
GYG2	-1.132219006	0.035413956
GOLGA6L5	-1.303623788	0.035481508
ZFP14	-1.066569657	0.035713203
ZNF408	-1.458536486	0.035869622
PDE6A	-1.340957326	0.035942584
LOC283501	-1.146835618	0.036081664
ACSS3	-1.008693695	0.036138024
SALL4	-1.849413011	0.036259533
GNAL	-1.055019361	0.036373951
POTEB	-1.26787212	0.036613892
CLEC6A	-1.279868745	0.036856162
OR2T4	-1.035695997	0.037049994
SNAR-E	-1.897741428	0.037196815
PATZ1	-1.257132457	0.037253527
LOC644242	-1.743565212	0.037322885
FGD5	-1.240715928	0.037463143
PFN1	-1.257936007	0.037514869
BOK	-1.218604856	0.037540523
LOC100188947	-1.088534235	0.037655911
MYH7B	-1.244046578	0.037690753
KCNK9	-1.161182866	0.038165798
MYCT1	-1.272275055	0.03827397
UXT	-1.513861126	0.038282263
LOC100128591	-1.041305882	0.038405122
LOC643401	-1.528228139	0.0387258
BMP3	-1.013460057	0.038853531
LOC728073	-1.300573896	0.03889099
LOC645586	-1.43688601	0.038987533
CNBD1	-1.211855153	0.039078868
MRGPRD	-1.110715153	0.039161634
RASSF6	-1.281287688	0.039274078
CPN2	-1.167607258	0.03934479
MKL2	-1.223852214	0.039385532
LOC388849	-1.136587231	0.039407217
PAK3	-1.673343832	0.039422863
LOC728558	-1.019858598	0.039438896
FLJ37035	-1.315717007	0.039650595
SLC8A2	-1.386795706	0.039743766
KRT78	-1.614367727	0.03980037
IRS4	-2.12484141	0.039902823
FLJ42200	-1.00833079	0.039927298
ZXDA	-1.244170965	0.039982862
ALDOC	-1.317916764	0.040164092
LOC644093	-1.099637818	0.040323141
TMEM229A	-1.422762432	0.040397645

GPR21	-1.076508006	0.040977333
MIR137HG	-1.141045028	0.040995612
LOC283728	-1.441907458	0.041039168
EBF2	-1.479257202	0.041048369
LOC100130876	-1.007045975	0.041148234
FLJ37505	-2.119185177	0.041158513
TREH	-1.078089427	0.041283436
PTPRS	-1.216979001	0.041295222
LCP1	-1.080456412	0.041339852
TMEM143	-1.025477531	0.0415218
CLEC18C	-1.310442251	0.04153398
NKD2	-1.117383439	0.041557352
LOC100128437	-1.674465702	0.041699145
PLA2G1B	-1.284625973	0.041723192
NFE4	-1.237926515	0.042026724
LOC284395	-1.015428604	0.042110967
FGFRL1	-1.687026463	0.042206987
TAF7L	-1.305145081	0.042381734
LRRC55	-1.274782579	0.042563845
LOC728196	-1.204850762	0.042813746
TNN	-1.513685557	0.043061888
POM121L4P	-1.806685189	0.043065169
LOC349196	-1.556257753	0.043070628
OR8K3	-1.10730766	0.043332963
JAKMIP3	-1.799744795	0.043643901
EML2	-1.422893416	0.043832637
GPR172B	-1.022380429	0.044035695
SNAR-G1	-1.197264038	0.04410191
POT1	-2.267171725	0.044127152
LOC553137	-1.026302163	0.044319814
LOC415056	-1.080834174	0.044678696
PRSS38	-1.286498692	0.044804054
DKK4	-1.017974887	0.044855969
COL6A2	-1.033899214	0.044895882
LOC100506514	-1.570264971	0.044902616
C1orf187	-1.397053624	0.04502353
ISLR2	-1.16409465	0.045111695
LOC339788	-1.223511987	0.045117902
ASCL4	-1.272019573	0.045216009
MATN	-1.24357793	0.045233225
DSCR10	-1.310870451	0.045292472
LOC100131792	-1.18393803	0.045341493
TCEB3B	-1.125130314	0.045887116
LOC100128402	-1.043301589	0.04615326
MAGEB10	-1.181040158	0.046334982
OR13H1	-1.118139456	0.046533185
LOC100652805	-1.183370456	0.046571714
DPY19L2P3	-1.286762701	0.046701518
BRD7	-1.15453395	0.04688221
LOC441956	-1.13498494	0.047262021
SMCR5	-2.084467745	0.047757312
LOC391767	-1.329142723	0.048017608
RABL2B	-1.281723221	0.048047275
SHOX2	-1.165353588	0.04813797
LOC157627	-1.140815816	0.048336564
USP17	-1.544554885	0.04875912
TUBB3	-1.056022156	0.048768946
MBD3L1	-1.135320811	0.048860296

LOC100131129	-1.491257954	0.048877599
PAX8	-1.070456908	0.049082517
HPSE2	-1.117748218	0.04909936
KRT222	-2.596636658	0.049224049
RN28S1	-3.269523552	0.049236383
LOC284014	-1.40771301	0.049808008
DEFB136	-1.031874436	0.049936131
PEG10	-1.579451597	0.049947281
LOC100505880	-1.332848007	0.049993873
