# Supplementary material for: Identification of Diagnostic Signatures and Immune Cell Infiltration Characteristics in Rheumatoid Arthritis by Integrating Bioinformatic Analysis and Machine-Learning Strategies
Source: Front Immunol. 2021 Oct 6;12:724934. doi: 10.3389/fimmu.2021.724934 (PMC8526926; doi:10.3389/fimmu.2021.724934)
Supplement: Supplementary Table 3 — The list of differentially expressed genes of GSE17755 [file Table_3.pdf]

Gene	logFC	P.Value
PHLDA3	1.199048656	9.25E-45
CEBPB	1.241452106	1.87E-43
NOC2L	1.054157968	4.71E-41
C21orf104	1.094668895	9.03E-39
C22orf30	1.153553841	5.46E-37
RNF157	1.30594419	6.29E-37
GABRD	1.133416011	9.98E-37
DEFA3	1.2308677	2.42E-35
ERAF	1.107269179	5.45E-34
LEPRE1	1.000367891	5.86E-34
C22orf24	1.09009361	6.05E-34
MEOX2	1.574764784	7.51E-34
NPHS2	1.126151175	3.32E-33
SCOC	1.380043429	4.10E-33
HOXA11	1.123622151	4.80E-33
EDA2R	1.195850945	3.65E-32
IL1R2	1.305042294	6.17E-32
EGR4	1.041559419	1.00E-31
NEK6	1.027950531	3.22E-31
EGR1	1.061155049	1.12E-28
CPB2	1.400530343	2.30E-28
CA1	1.002490805	5.38E-27
STXBP6	1.553299056	6.06E-27
OR2A9P	1.253087218	1.33E-26
MC5R	1.036810993	4.81E-25
PDE6H	1.089835256	1.01E-24
PIGL	1.035832445	6.26E-24
MTERF	1.242024573	4.80E-23
H19	1.018687584	1.10E-22
SYS1	1.015543407	1.36E-18
MYEF2	1.093735465	2.80E-18
S100A12	1.171833665	1.27E-16
HCFC2	2.029303789	1.69E-12
MAP4K5	-1.229498817	1.43E-45
C2orf16	-1.534706926	1.00E-43
PRKD2	-1.243991856	8.08E-38
LSP1	-1.387742234	2.86E-37
NNMT	-2.025751034	8.85E-36
SERPINB13	-1.115055352	1.18E-35
HOXA2	-2.574248181	1.49E-35
GNLY	-1.556435479	7.13E-34
SCN4B	-1.183116631	1.21E-32
ALDOC	-1.484393521	2.37E-32
CSRP2	-1.184666407	4.29E-31
IL32	-1.324543475	6.55E-31
DDX24	-1.002424471	5.18E-30
CLIP2	-1.096514219	3.82E-28
GHDC	-1.042260233	9.48E-28
MIER1	-1.180828161	1.74E-26
FAM64A	-1.297558349	1.29E-24
FAM95B1	-1.383160816	5.08E-23
DPCR1	-1.016706275	1.12E-18
ACTG1	-1.00541011	4.32E-17
IL26	-1.048712845	5.84E-16
ZFP14	-1.135568153	2.19E-14
HPSE2	-1.007651552	6.99E-11
MAT2B	-1.047445495	4.97E-10

CLSTN3	-1.201064654	8.80E-09
