# Supplementary material for: Identification of Diagnostic Signatures and Immune Cell Infiltration Characteristics in Rheumatoid Arthritis by Integrating Bioinformatic Analysis and Machine-Learning Strategies
Source: Front Immunol. 2021 Oct 6;12:724934. doi: 10.3389/fimmu.2021.724934 (PMC8526926; doi:10.3389/fimmu.2021.724934)
Supplement: Supplementary Table 4 — Fifty-four robust DEGs were screened with the RRA method from the two lists of differentially expressed genes [file Table_4.pdf]

Supplementary Table 4

IL32
MEOX2
OR2A9P
RNF157
CLIP2
HPSE2
EGR1
EGR4
H19
EDA2R
STXBP6
SCOC
SYS1
CLSTN3
FAM95B1
PIGL
S100A12
IL26
LEPRE1
C22orf30
ALDOC
PHLDA3
NOC2L
C22orf24
PDE6H
MYEF2
DDX24
GABRD
MAP4K5
MC5R
MIER1
HOXA11
LSP1
C21orf104
CPB2
C2orf16
FAM64A
DEFA3
MTERF
ZFP14
HOXA2
ACTG1
GHDC
DPCR1
NPHS2
SERPINB13
CSRP2
HCFC2
SCN4B
PRKD2
GNLY
NNMT
MAT2B
NEK6
